# Effect of Mechanical Recycling on the Crystallization of PA 11 and PA 11 LDPE Blends

**DOI:** 10.1002/marc.202500164

**Published:** 2025-03-29

**Authors:** Johanna Morales, Rose Mary Michell, Denis Rodrigue

**Affiliations:** ^1^ Department of Chemical Engineering Université Laval Quebec QC G1V 0A6 Canada; ^2^ Institut für Physik Martin‐Luther‐Universität Halle‐Wittenberg 06099 Halle Germany

**Keywords:** crystallization kinetics, isothermal crystallization, mechanical recycling, PA 11, thermal degradation

## Abstract

This study investigates the effect of mechanical recycling on the thermal crystallization of virgin polyamide (PA 11) and a post‐consumer PA 11 – low‐density polyethylene (LDPE) blend (90/10) over ten reprocessing cycles. Isothermal, non‐isothermal, and successive self‐nucleation and annealing (SSA) methods are used. Isothermal analysis revealed accelerated crystallization kinetics with increasing reprocessing cycles, as shown by an increase in the inverse of the half‐crystallization time (*1/τ_1/2exp_*) and a decrease in the crystallization energy barrier (*K_g_*), likely due to enhanced chain mobility and molecular weight reduction from thermal degradation. SSA analysis revealed differences in lamellar structures. After three cycles, virgin PA 11 presented a shoulder in the SSA profile, indicating the formation of thinner lamellae. In contrast, post‐consumer PA 11 showed a progressive increase in its main melting peak, suggesting the development of thicker lamellae with a more uniform molecular population. Thermogravimetric analysis showed reduced thermal stability, as indicated by lower activation energy (*E_a_*). Despite these changes, their effect is not significant to limit reprocessing, confirming their recyclability for at least ten cycles. To further assess their long‐term viability; structural, rheological, and mechanical properties will be presented in a subsequent study.

## Graphical abstract

The impact of mechanical recycling on the crystallization behavior of polyamide 11 and a post‐consumer PA 11 ‐ LDPE blend is studied over ten cycles. Isothermal analysis shows a lower energy barrier and an increase in the inverse of half‐crystallization time. SSA results indicate lamellar structural changes, while thermal stability slightly decreases. Both materials remain recyclable for at least ten cycles.

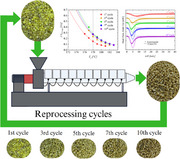

## 1 Introduction

Plastics significantly impact our modern life due to their versatility and diverse applications. However, their environmental effects, especially the accumulation of plastic waste and their contribution to global pollution, became a critical concern, driving the need for more sustainable and effective solutions. One solution is to use biobased polymers, such as polyamide 11 (PA 11), providing a sustainable alternative to fossil‐derived plastics and contributing to a circular economy through their recyclability. Despite these advantages, the mechanical recycling of biobased polymers, especially under extended thermal and mechanical exposure from multiple reprocessing cycles, remains an area requiring further investigation.

PA 11 is synthesized by the self‐polycondensation of 11‐aminoundecanoic acid, derived from castor oil, a renewable resource.^[^ ^1^, ^2^ ^]^ Furthermore, PA 11 is part of the long‐chain polyamides (LCP) family (more than 10 methylene groups present between the adjacent amide groups in the main chain).^[^ ^3^ ^]^ Compared with traditional polyamide 6 or 66 (PA 6 and PA 66), the production of this semi‐crystalline polymer emits ≈40% less carbon dioxide (CO_2_).^[^ ^4^ ^]^ Additionally, the lower number of amide groups per repeating unit in PA 11 contributes to its excellent thermal stability (decomposition temperature ≈430–455 °C),^[^ ^5^ ^]^ and mechanical properties (tensile modulus = 1600 MPa, tensile strength = 49 MPa, elongation at break = 40%).^[^ ^6^, ^7^, ^8^, ^9^ ^]^ PA 11 is also characterized by its exceptional resistance to chemicals and ultraviolet (UV) radiation, as well as lower water absorption.^[^ ^10^, ^11^ ^]^ These properties make PA 11 a versatile material for a wide range of applications, including automotive components, electrical insulations, and industrial parts.^[^ ^6^, ^12^, ^13^ ^]^ As a biobased polymer, PA 11 aligns with the growing emphasis on sustainability and circular economy, offering a renewable alternative to petroleum‐based polyamides without compromising performance.

The physical, chemical, and mechanical properties of semi‐crystalline polymers are highly influenced by their morphology, crystalline structure, and degree of crystallization.^[^ ^14^, ^15^, ^16^ ^]^ For instance, Vidakis et al.^[^ ^17^ ^]^ reported lower crystallinity of polyamide 12 (PA 12) (36.5% to 30.6%) after six cycles due to polymer chain scission during reprocessing. Colucci et al.^[^ ^18^ ^]^ investigated the effect of mechanical recycling on PA 66 composites reinforced with 30 wt.% carbon fibers. Their results showed a decrease in crystallinity from 26.2% to 24.6% after recycling due to polymer chain scission during the recycling process, resulting in shorter chains (reduced molecular weight).

Although several studies focused on the mechanical recycling of petroleum‐based polyamides (PA 6 and PA 66),^[^ ^15^, ^16^ ^]^ this work represents the first comprehensive investigation into reprocessing of biobased polyamide, PA 11. The study examines the effect of mechanical recycling on virgin PA 11 to compare the results with a post‐consumer waste (windmill blade wrapping): blend of PA 11 with low density polyethylene (LDPE). For each system, up to 10 reprocessing cycles are performed. However, to limit the amount of experimental work, the samples were characterized only for the 1^st^, 3^rd^, 5^th^, 7^th^, and 10^th^ cycles to determine the effect of reprocessing on the thermal properties as a first step.

## 2 Results

### 2.1 Non‐Isothermal Scanning

**Figure 1:**
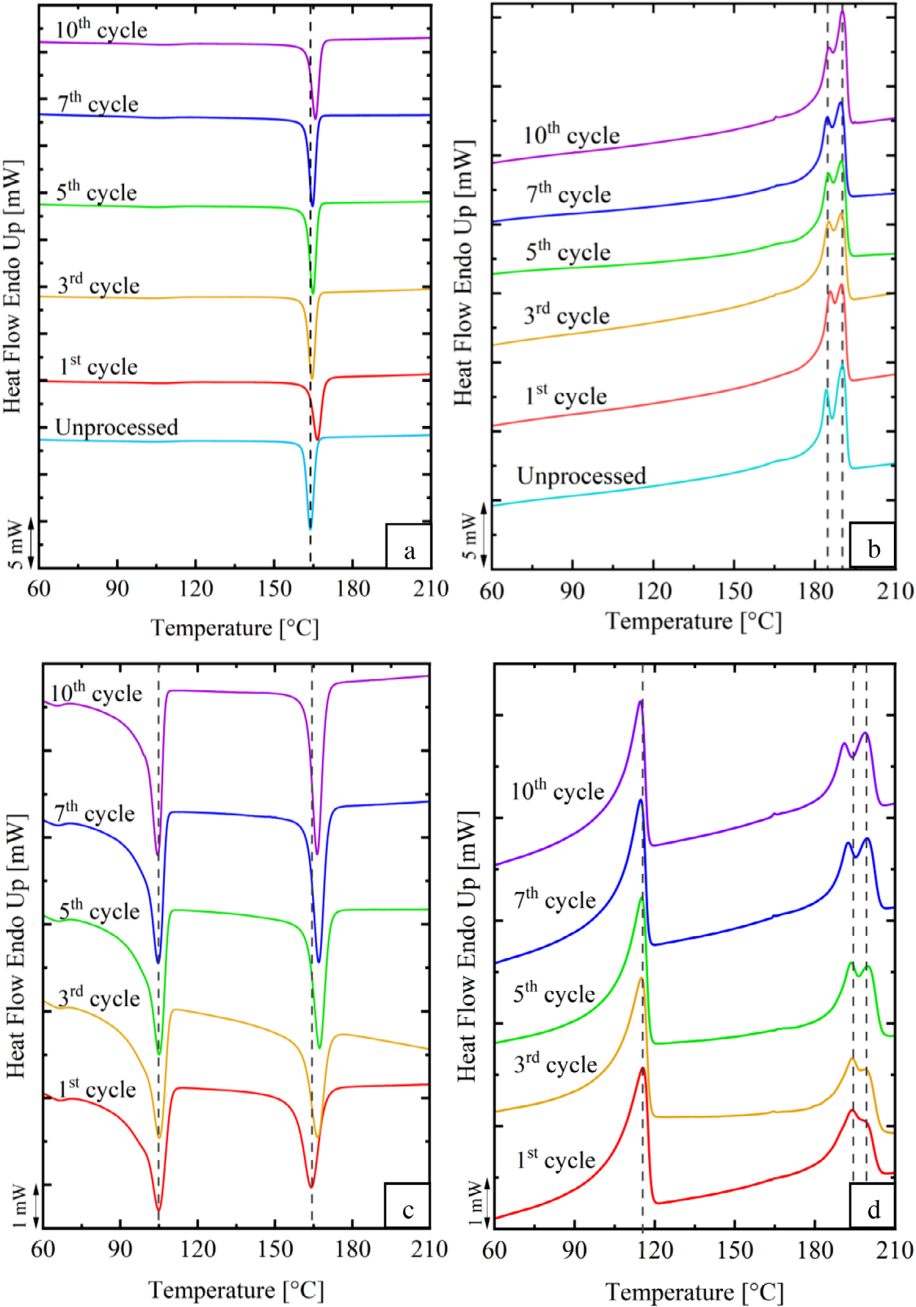
Differential scanning calorimetry curves: a) cooling and b) second heating scans for the virgin PA 11; c) cooling and d) second heating scans for the post‐consumer PA 11.

The influence of reprocessing on *T_c_* and *T_m_* on virgin and post‐consumer PA 11 was analyzed using DSC. **Figure** **1** presents the cooling and second heating curves for both materials and **Table** **1** summarizes the data obtained. For virgin PA 11, a single exothermic peak was observed during the cooling scans presented in Figure 1a. The peak remained stable, at 164 °C over ten reprocessing cycles. This stability indicates that reprocessing did not cause significant nucleation or structural changes in the crystalline regions. During the second heating scans, a characteristic double endothermic peak was observed presented in Figure 1b. The first peak at 184 °C corresponds to less ordered crystalline regions, while the second peak at 190 °C represents more stable and thermally ordered crystals. The second peak increased with successive reprocessing cycles, suggesting a melt‐recrystallization phenomenon. This behavior, commonly seen in polyamides, occurs when less ordered crystals (α' crystals form) melt and reorganize into more stable structures (α crystals form) during cooling and reheating.^[^ ^19^, ^20^, ^21^ ^]^ Despite ten reprocessing cycles being performed, the first and the second endothermic peaks remains almost constant. Su et al.^[^ ^22^ ^]^ reported similar results for PA 6; after sixteen reprocessing cycles, only the melt‐recrystallization process was observed.

**Table 1:** Thermal properties of the virgin PA 11 and post‐consumer PA 11.

Sample	Cycle	*T_c1_*	*T_c2_*	*ΔH_c1_*	*ΔH_c2_*	*T_m1_*	*T_m2_*	*ΔH_m1_*	*ΔH_m2_*	*X_c_*
		[°C]	[°C]	[J/g]	[J/g]	[°C]	[°C]	[J/g]	[J/g]	[%]
Virgin	Unprocessed	−	163.8	−	−47.8	−	184.1/190.1	−	51.8	52.7
	1^st^	−	166.5	−	−44.9	−	185.4/189.7	−	52.7	51.6
	3^rd^	−	164.4	−	−46.8	−	184.7/189.7	−	51.0	51.7
	5^th^	−	164.8	−	−46.5	−	184.7/189.7	−	52.1	52.2
	7^th^	−	164.6	−	−47.6	−	184.2/189.5	−	51.8	52.6
	10^th^	−	165.7	−	−47.3	−	184.8/190.1	−	52.5	52.8
	1^st^	104.8	164.1	−24.12	−52.2	115.5	193.8/199.4	50.2	23.2	63.7
Post‐consumer	3^rd^	105.0	166.5	−24.07	−50.6	114.9	193.9/199.9	51.9	25.6	63.8
	5^th^	104.9	167.3	−26.01	−51.9	115.1	193.4/199.9	50.0	25.9	63.4
	7^th^	104.6	166.9	−27.26	−52.7	114.7	192.0/199.4	49.4	26.6	63.6
	10^th^	104.4	166.3	−27.70	−53.2	114.6	190.6/198.7	49.9	27.1	64.2

For post‐consumer PA 11 a more complex thermal behavior was observed. Two distinct exothermic peaks were observed during the cooling scans (Figure 1c); the first peak at 104 °C, corresponding to low density polyethylene (LDPE), and the second peak at 164 °C attributed to PA 11. The second heating scans (Figure 1d) showed an endothermic peak at 115 °C for LDPE and a double endothermic peak for PA 11 at 193 and 199 °C, respectively. The lower exothermic and endothermic peaks values are characteristic of LDPE and align with its known thermal properties.^[^ ^23^, ^24^ ^]^ The absence of significant changes in the thermal properties over all cycles aligns with previous studies on aliphatic polyamides,^[^ ^16^ ^]^ indicating that chain scission during reprocessing mainly affects the mechanical properties rather than thermal stability.

### 2.2 Isothermal Crystallization

**Figure 2:**
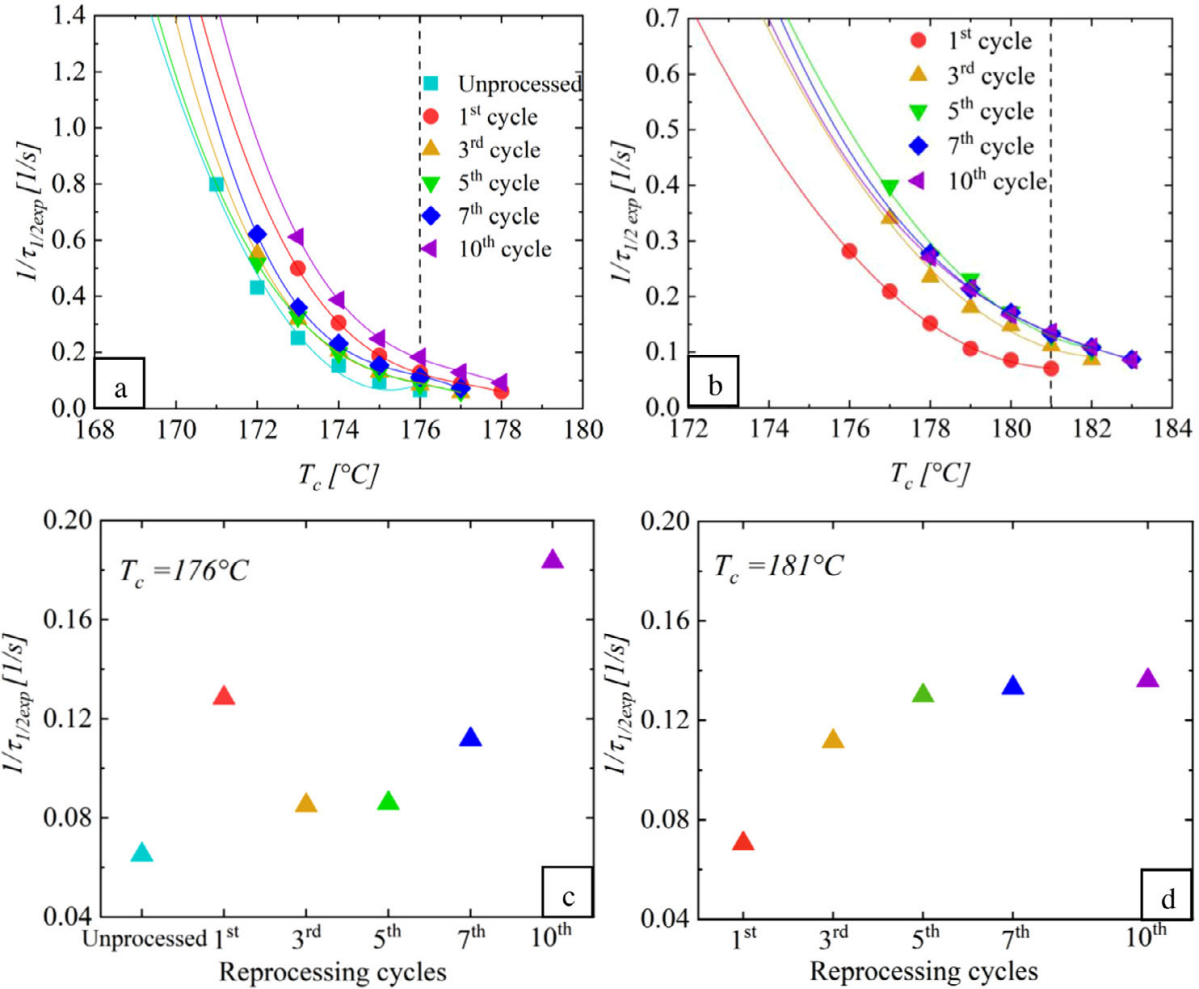
Values of *1/τ_1/2exp_* as a function of the isothermal crystallization temperature (*T_𝑐_*) for: a) virgin PA 11, b) post‐consumer PA 11, c) virgin PA 11 at *T_c_* = 176 °C and d) post‐consumer PA 11 at *T_c_* = 181 °C.

The effect of reprocessing on the crystallization rate of virgin and post‐consumer PA 11 was also investigated. **Figure** **2**a,b present the overall crystallization rate of virgin and post‐consumer PA 11 as a function of the crystallization temperature (*T_c_*). For every *T_c_*, as reprocessing cycles increase, the inverse of the half‐crystallization time (*1/τ_1/2exp_*) values for both materials also increase. This behavior is best illustrated in Figure 2c,d, where an increase in *1/τ_1/2exp_* is observed with successive reprocessing cycles at a constant *T_c_* (176 °C for virgin PA 11 and 181 °C for the post‐consumer material). The curves in Figure 2a,b are similar to the right‐hand side of a bell and reflect the interaction between nucleation and crystal growth kinetics. At lower *T_c_*, increased supercooling promotes rapid nucleation despite limited chain mobility, resulting in higher *1/τ_1/2exp_* values. In contrast, at higher *T_c_*, the reduced supercooling decreases nucleation, extending the crystallization process and leading to lower *1/τ_1/2exp_* values. The results presented in Figure 2c,d, show that reprocessing increases the crystallization rate, suggesting that less energy is required for crystallization due to improved chain mobility, which is attributed to a reduction in molecular weight. This observation aligns with studies by Yap et al.^[^ ^25^ ^]^ and Lee et al.^[^ ^26^ ^]^ suggesting that higher *1/τ_1/2exp_* can be associated with lower molecular weight in the reprocessing of PA 6 up to five and three cycles, respectively.

Avrami's theory is often used to model the isothermal crystallization kinetics by using the overall crystallization rate. In this work, the experimental protocols and analyses established by Lorenzo et al.^[^ ^27^, ^28^ ^]^ were used. The Avrami equation can be written as:

(1) $$1-V_{c}\left(t\right)=exp\left(-kt^{n}\right)$$

where 𝑉_𝑐_ is the relative volumetric transformed fraction (mg/mg), *n* is the Avrami index (‐), and *k* is the overall crystallization rate constant (min^−𝑛^) containing contributions from nucleation and growth.^[^ ^27^, ^29^ ^]^ The parameters *n* and *k* can be obtained from the slope and intercept of the Avrami plot as *log [−ln (1 − X𝑡)]* versus *log(t)* (where *X𝑡* is the relative crystallinity as a function of time).

To further analyze the isothermal crystallization kinetics of virgin PA 11, the Avrami's theory was used. Typical curves of this model (Figure S1, Supporting Information) show a strong fit between the model and experimental data, especially at low crystallinity levels (up to 50%) across all reprocessing cycles. These findings confirm that the primary crystallization stage is well‐captured by this model. Furthermore, the increase in *k* values (Table S1, Supporting Information) follows the experimentally observed trend and are associated with enhanced chain mobility and molecular weight reduction caused by chain scission during thermal degradation. These structural changes accelerate the crystallization process, as observed in other semi‐crystalline polymers, such as PA 6 and PA 66.^[^ ^30^, ^31^, ^32^, ^33^ ^]^ The increase of *1/τ_1/2exp_* and *k* values show the impact of thermal degradation in crystallization kinetics during mechanical recycling.

For post‐consumer PA 11, the Avrami model also provides a good fit to the experimental data (Figure S2, Supporting Information). Similarly to virgin PA 11, the theoretical *k* values (Table S2, Supporting Information) increase with the number of reprocessing cycles, confirming that the observed trend is consistent for both materials.

The *n* values provide information about the nucleation type and the dimensionality of crystal growth. Theoretically, it is composed of two terms: *n = n_d_ +_n_*, where *n_d_* corresponds to the dimensionality of crystal growth (2 for axial growth and 3 for spherulitic growth), while *n_n_* represents the time dependence of nucleation (0 for instantaneous and 1 for sporadic nucleation). In general, polymers crystallizing in a spherulitic morphology have *n =* 4 when nucleation is sporadic (low supercooling) and *n =* 3 when nucleation is instantaneous (high supercooling). Intermediate values between 3 and 4 suggest a transition between these nucleation mechanisms.^[^ ^27^ ^]^ The values of *n* obtained in this study, as shown in Tables S1 and S2 (Supporting Information), range between 3 and 4. The results show that as *T_c_* increases, the values of *n* also increase to ≈3 for virgin and 4 for post‐consumer PA 11. Changes in *n* indicate that the conditions, such as the molecular weight distribution and crystallization temperatures, strongly influence the crystallization mechanism of PA 11. However, no clear trend is observed with respect to the number of reprocessing cycles.

The Lauritzen–Hoffman (L─H) theory has been widely used to investigate crystal growth in polymers.^[^ ^14^, ^29^, ^34^, ^35^, ^36^ ^]^ In the L─H theory, crystal development involves two steps: the formation of secondary nuclei at the growth face and the subsequent growth of the polymer crystal along these nucleated regions. As polymer chain accumulate on the growth face, the resulting structure advances, forming well‐defined crystalline lamellae. Although the dimensions of these nuclei are important in theoretical research, their physical measurement is limited by experimental resolution. Consequently, the L─H theory provides a valuable method to analyze secondary nucleation and crystal growth in polymers.

Theoretically, the growth rate is influenced by molecular weight, local motion transport, and secondary nucleation. The relationship between these factors can be expressed as:^[^ ^35^, ^36^, ^37^ ^]^

(2) $$\frac{1}{\tau_{\frac{1}{2}}}\left(T\right)={\left(\frac{1}{\tau_{\frac{1}{2}}}\right)}_{0}\;\exp\left(\frac{-U^{*}}{R\left(T_{c}-T_{\infty }\right)}\right)exp\left(\frac{-K_{g}}{T_{c}\Delta Tf}\right)$$

where ${\left(\frac{1}{\tau_{\frac{1}{2}}}\right)}_{0}$ is a crystallization rate constant for the overall crystallization, U* is the activation energy for the transport of the chains to the growing front (≈1500 cal mol^−1^), *R* is the gas constant (8.314 J/(mol K)), *T_c_* is the isothermal crystallization temperature, *T_∞_* is the temperature at which chain mobility ceases, usually taken as *T_g_* − 30 K (*T_g_* = 43 °C in this study), *ΔT* is the supercooling value defined as $\left(T_{m}^{0}-T_{c}\right)$, $\;T_{m}^{0}$ is the equilibrium melting temperature (476 K),^[^ ^29^ ^]^ *f *is a temperature correction term $\left(\frac{2\;T_{c\;}}{T_{m}^{0}+T_{c}}\right)$, and *K_g_* is the energy barrier for the crystallization process of the overall nucleation and crystal growth kinetics (K^2^). The results are presented in Figure S3 (Supporting Information) and the numerical data are presented in **Table** **2**.

**Table 2:** Kinetics parameters for virgin PA 11 and post‐consumer PA 11 obtained by Equation (2) (L─H model).

Sample	Cycle	*K_g_*
		[K^2^]
Virgin	Unprocessed	2.17 × 10^5^
	1^st^	1.58 × 10^5^
	3^rd^	1.83 × 10^5^
	5^th^	1.76 × 10^5^
	7^th^	1,71 × 10^5^
	10^th^	1.43 × 10^5^
Post‐consumer	1^st^	8.71 × 10^4^
	3^rd^	7.53 × 10^4^
	5^th^	7.43 × 10^4^
	7^th^	6.66 × 10^4^
	10^th^	6.28 × 10^4^

To understand the effect of reprocessing on crystal growth in PA 11, the crystallization kinetics were analyzed using L─H theory. The values of *K_g_* for virgin and post‐consumer PA 11 are presented in Table 2. The values for the virgin material show a decrease from 2.17 × 10^5^ K^2^ for unprocessed sample to 1.43 × 10^5^ K^2^ after the 10^th^ cycle. Similarly, for the post‐consumer PA 11, the values of *K_g_* present a decrease over all reprocessing cycles, from 8.71 × 10^4^ K^2^ for the 1^st^ cycle to 6.28 × 10^4^ K^2^ for the 10^th^ cycle. This reduction reflects the cumulative effect of reprocessing, leading to chain scission and a consequent reduction in molecular weight.^[^ ^32^ ^]^ As a result, the enhanced mobility of shorter chains lowers the energy barrier for chain folding, promoting the formation of thinner lamellae.

### 2.3 Successive Self‐nucleation and Annealing (SSA)

The SSA technique is a well‐established thermal fractionation method exploiting the molecular segregation capability of semi‐crystalline polymer systems subjected to isothermal crystallization or annealing.^[^ ^38^ ^]^ This theory was thoroughly explained in previous works.^[^ ^38^, ^39^, ^40^, ^41^, ^42^, ^43^, ^44^ ^]^ SSA is based on the self‐nucleation (SN) concept introduced by Fillon et al.^[^ ^45^ ^]^ and developed by Muller et al.^[^ ^42^ ^]^ This thermal method enhances nucleation density in polymers by creating self‐seeds or self‐nuclei. The process involves erasing the polymer's crystalline memory, establishing a controlled semi‐crystalline state, and thermally conditioning the sample at specific self‐nucleation ideal temperatures (*T_s_*) to promote self‐nucleation.

In general, SN is characterized by controlled heating and cooling rates, which can be divided in three distinct domains:

1. Domain I (complete melting): In this domain, the polymer undergoes complete melting, which means that the crystalline structure is completely erased; i.e., no nucleation from previous structures influences the crystallization process and *T_c_* is unaffected by prior crystallization.
2. Domain II (self‐nucleation): This domain is marked by an increase in crystallization temperature (*T_c_*) without annealing, where the ideal self‐nucleation temperature (*T_s, ideal_*) is located and represents the maximum nucleation density.
3. Domain III (nucleation and annealing): This domain is marked by an increase in crystallization temperature (*T_c_*) with annealing.

**Figure 3:**
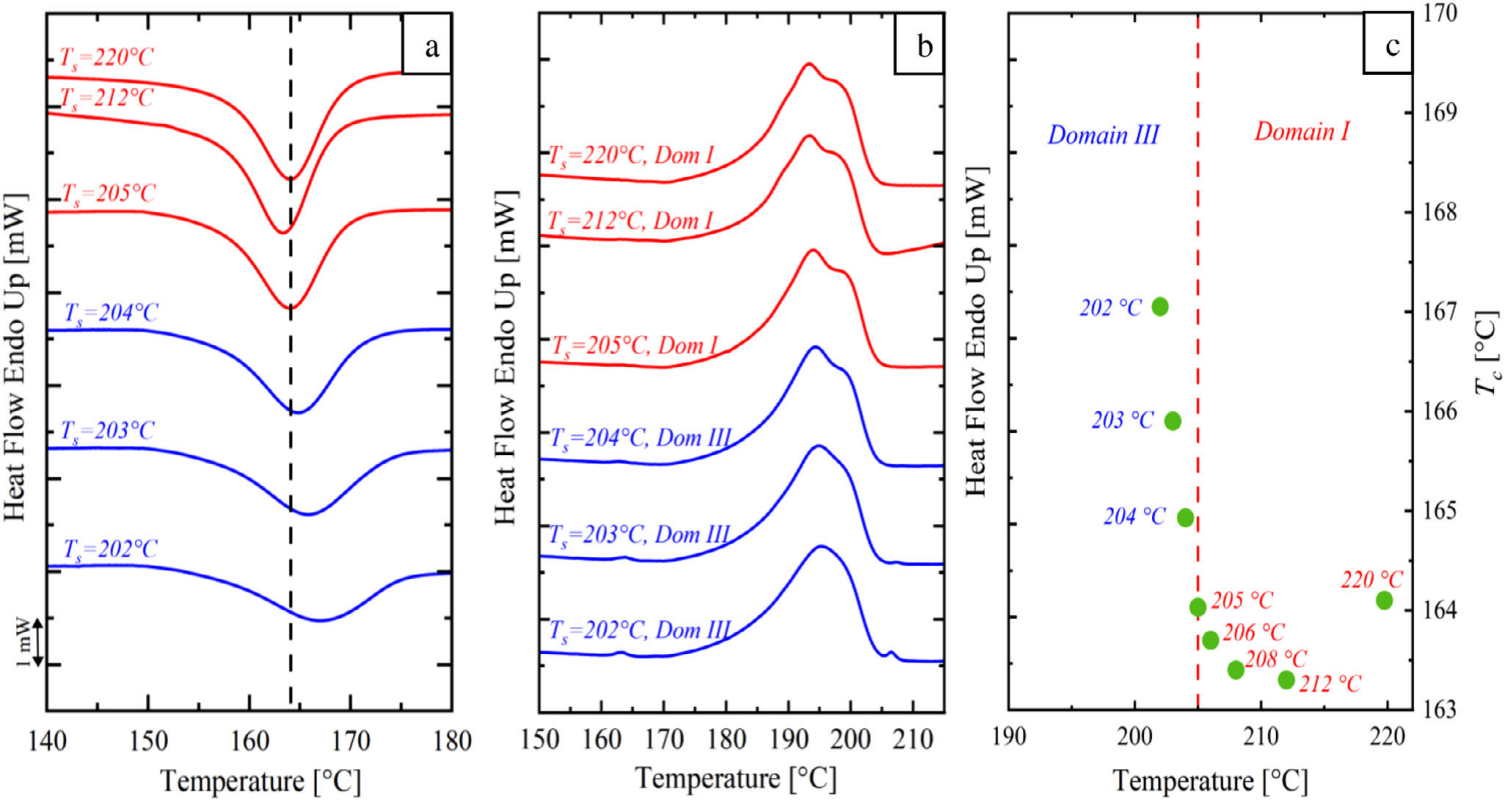
DSC results: a) cooling scans (10 °C min^−1^) for post‐consumer PA 11 after 5 min at *T_s_*; b) subsequent heating scans (10 °C min^−1^) after the cooling runs; c) representation of the self‐nucleation domains for PA 11.

These domains are identified through DSC analyses. For example, **Figure** **3** shows the SN analysis used to determine *T_s_, _ideal_* for the post‐consumer PA 11. Typically, *T_s_* is within domain II. However, some polymers, such as the PA 11 studied here, transition directly from domain I to domain III due to high heterogeneous nuclei density.^[^ ^46^ ^]^ For the post‐consumer PA 11, this transition occurs at 204 °C, as evidenced by a small peak in Figure 3b. Therefore, *T_s_, _ideal_* = 205 °C was selected which is located just below domain I, but close to the beginning of domain III. Figure 3c illustrates the self‐nucleation domains on top of the standard melting endotherm of PA 11. The plot also shows how *T_c_* changes as a function of *T_s_*. In domain I, *T_c_* remains constant, while it significantly increases in domain III, as expected.^[^ ^38^, ^43^ ^]^

**Figure 4:**
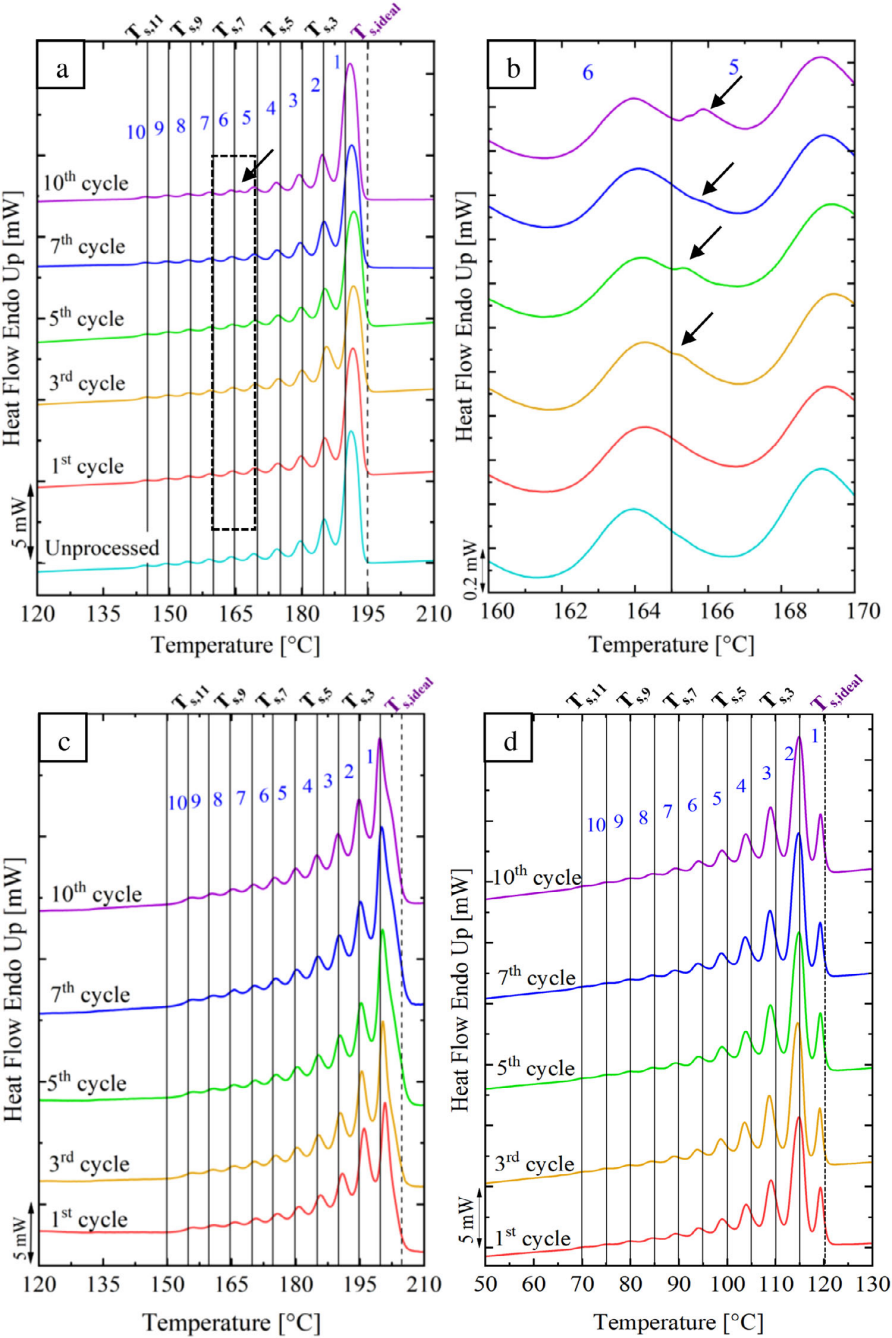
Final heating scans after SSA fractionation for: a) virgin PA 11, b) magnification of the peaks 5 and 6 for the virgin PA 11, c) post‐consumer PA 11, and d) LDPE in the post‐consumer PA 11.

The final DSC heating runs after SSA fractionation for the virgin PA 11, post‐consumer PA 11, and LDPE in the post‐consumer blend are presented in **Figure** **4**. The melting point distributions for both virgin and post‐consumer PA 11 confirm a monomodal distribution of chain lengths characteristic of linear polyamides.^[^ ^41^ ^]^ Each DSC peak represents the melting of crystals with distinct lamellar thicknesses, reflecting the molecular structure of the polymer. Virgin PA 11 exhibits more uniform lamellae thickness than post‐consumer PA 11 (Figure 4a). In linear homopolymers, such as PA 11, fractionation results from differences in molecular weight, as the chain length influences the melting behavior.^[^ ^44^ ^]^ Higher melting points correspond to thicker lamellae, with the thickest lamellae formed by the longest polymer chains.^[^ ^47^ ^]^ In virgin PA 11, the unprocessed sample exhibited 10 distinct melting peaks after SSA fractionation. The most prominent fraction (*T_m_* = 191 °C) represents the thickest lamellae formed with *T_s_, _ideal_* = 195 °C and subsequent annealing. Additionally, a small shoulder is observed in the 3^rd^ reprocessing cycle which increases with more reprocessing as shown in Figure 4b. This can be attributed to thinner lamellae formed by shorter chain segments.

**Figure 5:**
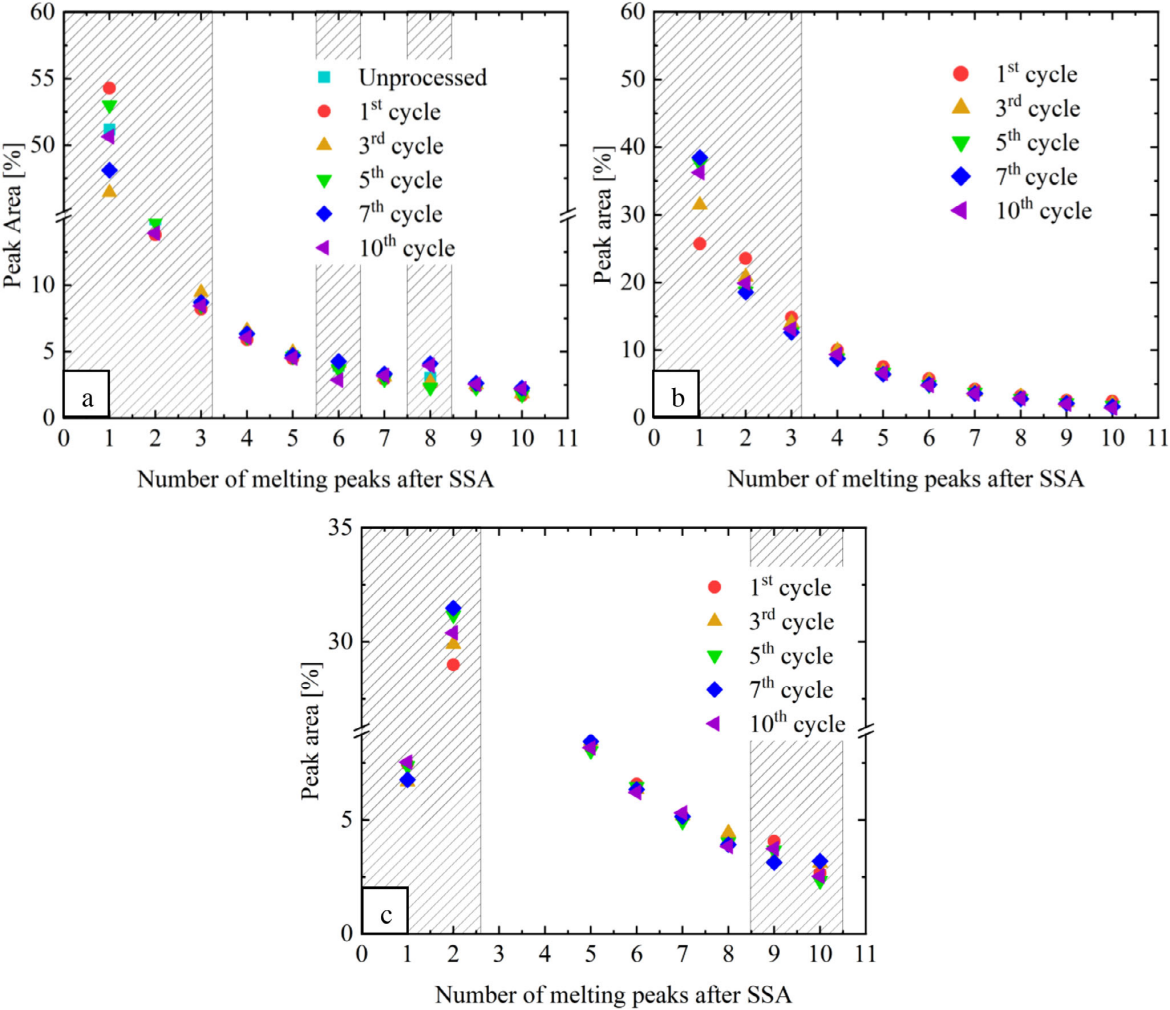
Peak area after SSA fraction for: a) virgin PA 11, b) post‐consumer PA 11, and c) LDPE in the post‐consumer PA 11.

Post‐consumer PA 11 does not present extra peaks or shoulders (Figure 4c) during reprocessing cycles. However, it shows a marked increase in the relative content of melting peak 1 with successive reprocessing cycles, increasing from 25% to 36% by the 10^th^ cycle (Figure 5b). Simultaneously, peaks 2 and 3 decreased from 23% to 19% and 15% to 13%, respectively. This trend suggests an increase in thickness corresponding to the most regular molecular populations, probably driven by chain scission generating more chain ends and facilitating lamella formation.^[^ ^48^ ^]^ LDPE present in the blend does not show a change in the SSA fractionation (Figure 4d). However, an increasing trend in the relative content of the melting peak 2 is clearly observed (**Figure** **5**c) associated with chain scission, which is in agreement with the results of post‐consumer PA 11.

### 2.4 Thermogravimetric Analysis (TGA)

**Figure 6:**
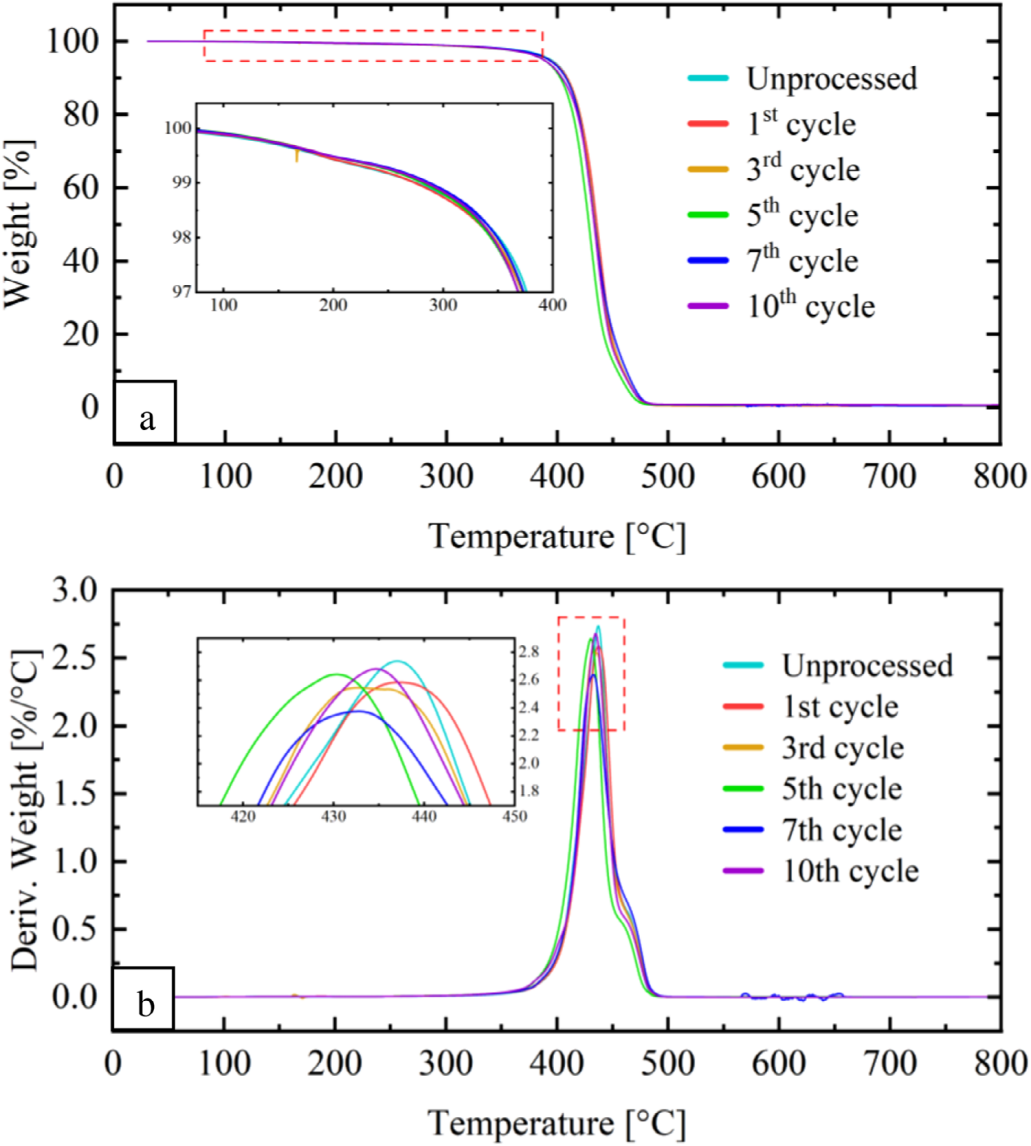
TGA curves for the virgin PA 11: a) weight as a function of temperature and b) the derivatives (DTG).

The analysis of the thermal stability of PA 11 during reprocessing is important to evaluate the recyclability and potential degradation mechanisms. **Figure** **6** presents the TGA and derivative thermogravimetric analysis (DTG) curves for virgin PA 11 samples. A minor mass loss ≈100 °C was observed in the TGA curves (Figure 6a) which is attributed to water (humidity) desorption. Thermal decomposition occurs in a single step, as indicated by the monomodal DTG curves (Figure 6b) consistent with previous studies.^[^ ^49^, ^50^ ^]^ The maximum degradation temperature (*T_p_ I*) for virgin PA 11, derived from the DTG peak, is summarized in **Table** **3**. A slight decrease in *T_p_ I* is observed with successive reprocessing cycles, decreasing from 437 °C (unprocessed) to 434 °C (10^th^ cycle). This slight decrease suggests thermal degradation associated with chain scission during reprocessing, leading to lower molar mass. As the molar mass decreases, less energy is required for thermal decomposition, in agreement with findings on PA 6 by Crespo et al.^[^ ^31^ ^]^ and Domingo and Souza^[^ ^51^ ^]^ who observed a decrease in *T_p_ I* after five and seven reprocessing cycles, respectively.

**Table 3:** TGA results for the virgin and post‐consumer PA 11.

Sample	Cycle	*T_onset_*	*T_endset_*	*T_p_ I*	*T_p_ II*	Total loss
		[°C]	[°C]	[°C]	[°C]	(%)
Virgin	Unprocessed	409.1	462.2	437.0	−	99.6
	1^st^	408.1	462.2	437.4	−	99.6
	3^rd^	408.1	460.3	434.1	−	99.6
	5^th^	404.4	454.6	425.4	−	99.5
	7^th^	406.3	462.2	432.5	−	99.5
	10^th^	406.3	460.3	434.5	−	99.3
Post‐consumer	1^st^	418.5	484.7	454.8	473.3	99.0
	3^rd^	417.6	484.7	456.0	474.4	99.1
	5^th^	418.5	489.5	458.7	476.6	99.2
	7^th^	418.5	489.5	459.7	476.1	99.2
	10^th^	418.5	489.5	456.9	476.3	99.0

**Figure 7:**
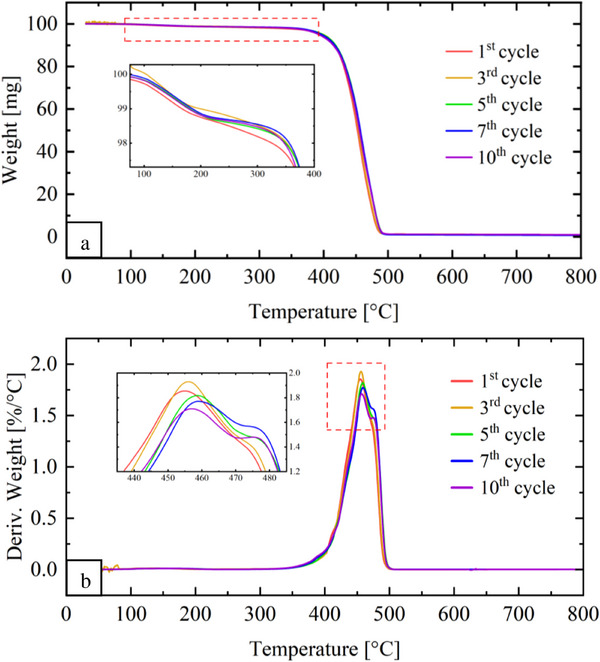
TGA curves for the post‐consumer PA 11: a) weight as a function of temperature and b) the derivatives (DTG).

The TGA and DTG curves of the post‐consumer PA 11 samples are presented in **Figure** **7**. As observed for virgin PA 11, a mass loss ≈100 °C was attributed to the water desorption (Figure 7a). However, DTG curves for the blends are bimodal, indicating separate degradation process for the components (Figure 7b). The first DTG peak corresponds to PA 11 (*T_p_ I*), while the second peak is attributed to LDPE (*T_p_ II*). A similar process was reported by Yordanov and Minkova^[^ ^52^ ^]^ in the TGA of PA 6‐LDPE blends. *T_p_ I* and *T_p_ II* values are summarized in Table 3, indicating a slight increase in the thermal stability with successive reprocessing cycles. For PA 11, *T_p_ I* increases from 454 to 456 °C, while *T_p_ II* increases from 473 to 476 °C for LDPE. However, no significant changes are observed in the temperature of both materials, indicating that the samples remain thermally stable even after ten reprocessing cycles.

To further evaluate the effect of reprocessing on the thermal stability of PA 11, *E_a_* of thermal degradation was determined using the Friedman method. This method provides insights into the energy barrier required for decomposition, which is closely linked to changes in molecular structure caused by repeated processing.^[^ ^53^ ^]^ The kinetic parameters for the thermal degradation of all samples were calculated using Equation (3) as:

(3) $$ln\;\left(\frac{d\alpha }{dt}\right)=\ln\left(Af\left(\alpha \right)\right)\;-\frac{E_{a}}{RT}$$

The activation energy (*E_a_*) was determined by plotting $ln(\frac{d\alpha }{dt})\;$ as a function of 1/T, where the slope corresponds to − *E_a_*/*R* and the intercept to ln(Af(α)).^[^ ^54^ ^]^

The *E_a_* values for virgin and post‐consumer material are presented in **Table** **4**. For virgin PA 11, the calculated *E_a_* for the unprocessed sample is 222 kJ mol^−1^, similar to previously reported values (249 kJ mol^−1^).^[^ ^49^ ^]^ A clear decreasing trend in *E_a_* is observed with reprocessing: from 222 kJ mol^−1^ for the unprocessed sample to 200 kJ mol^−1^ after the 10^th^ cycle. Lower *E_a_* reflects a reduction in thermal stability, consistent with the slight decrease in *T_max_* observed in the TGA results (Figure 6a). For post‐consumer PA 11, a similar pattern is observed, with *E_a_* decreasing from 164.8 kJ mol^−1^ in the first cycle to 144 kJ mol^−1^ by the 10^th^ cycle. Lower *E_a_* for both materials suggests that chain scission during reprocessing decreases the energy barrier for thermal decomposition, corroborating the degradation trends from thermal analysis, discussed above.

**Table 4:** Kinetic parameters (TGA) of the virgin and post‐consumer PA 11.

Sample	Cycle	*Ea*	*Ln(A)*	*R^2^*
		[kJ/mol]		
Virgin	Unprocessed	222.7	31.12	0.995
	1^st^	220.4	30.67	0.996
	3^rd^	213.9	29.63	0.990
	5^th^	214.0	29.93	0.994
	7^th^	212.8	29.46	0.991
	10^th^	200.9	27.47	0.993
Post‐consumer	1^st^	164.8	20.55	0.997
	3^rd^	164.8	20.45	0.995
	5^th^	154.3	18.65	0.996
	7^th^	149.8	17.91	0.997
	10^th^	144.0	17.00	0.995

## 3 Conclusion

This study evaluated the effect of mechanical recycling on the crystallization and thermal behavior of virgin PA 11 and a post‐consumer PA 11‐LDPE blend over ten reprocessing cycles. Isothermal analysis showed that reprocessing accelerated crystallization kinetics, as observed by the increase in *1/τ_1/2exp_* and the decrease in energy barrier for the crystallization process (*K_g_*). This behavior indicated that shorter crystallization times where required for both materials, attributed to increased chain mobility and reduced molecular weight due to chain scission during thermal degradation. SSA analysis revealed differences in the lamellar structure of virgin and post‐consumer PA 11 during reprocessing. Virgin PA 11 presented a shoulder in the SSA profile after the 3^rd^ cycle, which became more pronounced with successive cycles, related to thinner lamellae. In contrast, post‐consumer PA 11 showed a progressive increase in the relative content of its main melting peak, suggesting a shift toward thicker lamellae attributed to more regular molecular populations. LDPE in the blend showed no significant change in its SSA fractionation profile but presented an increasing trend in one of its melting peaks, supporting the influence of chain scission on the thermal behavior of the post‐consumer material. Thermogravimetric analysis showed that reprocessing reduced the thermal stability of virgin and post‐consumer PA11, as evidenced by decreased *E_a_*. However, from a thermal perspective, no significant changes were observed regarding temperature, processing factors, or application conditions. Future analyses will focus on other properties such as: structural, rheological, and mechanical to determine the maximum number of cycles that PA 11 can undergo before its properties are substantially compromised.

## 4 Experimental Section

### Materials

The virgin PA 11 used was RILSAN BESNO TL provided by Arkema (USA) with a density of 1020 kg m^−3^ and a melt flow index (MFI) of 1 g/10 min (2.16 kg, 235 °C).^[^ ^55^ ^]^ The post‐consumer PA 11 was a blend of PA 11 with LDPE (90/10) and supplied by MRC La Haute‐Gaspésie (Canada) coming from windmill packaging (blades wrapping). The LDPE content in the blend was determined from the deconvoluted derivative TGA curves presented in Figure S4 (Supporting Information).

### Preparation of Post‐Consumer Material

The post‐consumer PA 11 was received as films, then cut and reprocessed in a co‐rotating twin‐screw extruder (Leistritz ZSE‐27, Germany) with a length/diameter (L/D) ratio of 40 and 10 heating zones (die diameter = 2.7 mm). The total flow rate was 0.5 kg/h, and the screw speed was set at 70 rpm. Since PA 11 has a melting temperature (*T_m_*) ≈190 °C,^[^ ^56^ ^]^ the temperature profile was set as: 175 °C for the first zone (feed), 210 °C for the second zone, 220 °C for the third zone, 235 °C for the fourth to the eighth zone, 225 °C for the ninth zone, and 195 °C for the tenth zone (die) to avoid degradation. The glass transition temperature (*T_g_*) of PA 11 ranges between 45 and 55 °C. Therefore, the extrudates were cooled using tap water at room temperature (20–25 °C). Finally, the filament was pelletized using a Conair model 304 pelletizer (Conair, USA) and then oven‐dried for 4 h at 80 °C to remove moisture.

### Reprocessing Cycles

The samples were reprocessed in the same co‐rotating twin‐screw extruder up to 10 times with the same conditions as mentioned above. Samples were taken after the 1^st^, 3^rd^, 5^th^, 7^th^, and 10^th^ reprocessing cycles for analysis. This methodology was used for virgin and post‐consumer materials. Finally, the samples were oven‐dried for 4 h at 80 °C to eliminate residual moisture.

### Characterization—Non‐Isothermal Scanning

The samples were analyzed on a DSC‐8 from Perkin Elmer (USA). The data collected were analyzed using the Pyris software version 11. Samples of ≈5 mg were sealed in aluminum pans and all the experiments were performed under a N_2_ atmosphere (flow rate = 20 mL min^−1^). For the non‐isothermal scanning, the samples were first melted at 220 °C and the thermal history was erased by holding the temperature for 3 min before cooling down from the melt state to 0 °C at a rate of 10 °C min^−1^. Finally, the samples were heated back to 220 °C at 10 °C min^−1^. The degree of crystallinity (*X_𝑐_*) was calculated as:

(4) $$X_{c}=\left(\frac{\Delta H_{m}}{\Delta H_{m}^{0}}\right)\;\left(\frac{1}{w}\right)\left(100\%\right)$$

where $\Delta H_{m}$ is the experimental heat of fusion (J/g), $\Delta H_{m}^{0}$ is the theoretical heat of fusion of the 100% crystalline PA 11 (189.05 J/g),^[^ ^57^ ^]^ and *w* is the weight fraction (wt/wt) of PA in the samples.

### Characterization—Isothermal Crystallization

After erasing the thermal history for 3 min at 220 °C, samples (≈5 mg) were rapidly cooled down from the melt state at 80 °C min^−1^ to the desired crystallization temperature. The heat flux was recorded as a function of time until equilibrium was achieved (about three times the half‐crystallization duration). Then, the samples were heated at a rate of 20 °C min^−1^ to record the melting behavior of the isothermally crystallized polymer. Preliminary tests were performed to identify the appropriate crystallization temperatures (*T_𝑐_*). The samples were rapidly cooled down from the melting point at 80 °C min^−1^ to a predetermined *T_𝑐_* and then heated at 20 °C min^−1^ to determine if any melting could be observed. Then, a test with a more precise *T*_𝑐_ was carried out until no crystallization occurred during the previous cooling. A *T_𝑐_* temperature range was used, including at least six different temperatures.^[^ ^27^ ^]^

### Characterization—Successive Self‐Nucleation and Annealing (SSA)

Samples (≈5 mg) were used to conduct the SSA tests and a detailed methodology can be found in previous works.^[^ ^38^, ^39^, ^41^, ^42^, ^43^, ^58^, ^59^ ^]^ After erasing the thermal history, the samples were cooled from the melting point of 220 to 0 °C at a rate of 50 °C min^−1^ to produce a standard thermal history. The samples were then heated at 50 °C min^−1^ until they reached the initial self‐seeding temperature or *T_s_* (virgin = 195 °C and post‐consumer = 205 °C). The holding time at *T_s_*, or fractionation time, was fixed at 5 min. Then, the samples were cooled at a rate of 50 °C min^−1^ to a temperature sufficiently low to allow for crystallization at 0 °C. Subsequently, the sample was heated once again at 50 °C min^−1^, but this time the temperature (*T_s_*) was set at 5 °C below the first one. This indicates that a fractionation window of 5 °C was used; i.e., the temperatures (*T_s_*) were separated by 5 °C during the heating and cooling cycles. The process was repeated until the complete melting range of the polymer was covered, followed by a final heating scan to melt all the produced thermal fractions.

### Characterization—Thermogravimetric Analysis (TGA)

Samples of 5–10 mg were used for the study. The weight curves were recorded using a TA Instruments (USA) model Q5000IR from 20 to 700 °C at a heating rate of 10 °C min^−1^ under a nitrogen atmosphere (25 mL min^−1^).

### Ethical Statement

This article does not contain any studies with human or animal subjects performed by any of the authors. Ethical approval was not required.

## Conflict of Interest

The authors declare no conflict of interest.

## Author Contributions

J.M. performed conceptualization (lead); investigation (lead); data curation (lead); visualization (lead); wrote the original draft preparation (lead); formal analysis (equal); methodology (equal); wrote the original draft and edited (equal). R.M.M. performed supervision (supporting); wrote the original draft and edited (equal); methodology (equal); formal analysis (equal); conceptualization (equal). D.R. performed supervision (lead); conceptualization (supporting); funding acquisition (lead); resources (lead); wrote the original draft and edited (equal).

## Supporting information



Supporting Information

## Data Availability

The data that support the findings of this study are available in the supplementary material of this article.
