# Supplementary material for: Effect of Mechanical Recycling on the Crystallization of PA 11 and PA 11 LDPE Blends
Source: Macromol Rapid Commun. 2025 Mar 29;46(23):2500164. doi: 10.1002/marc.202500164 (PMC12687675; doi:10.1002/marc.202500164)
Supplement: Supplementary file 1 — Supporting Information [file MARC-46-2500164-s001.docx]

Supporting Information

**Title:** Effect of mechanical recycling on the crystallization of PA 11 and PA 11 - LDPE blends

*Johanna Morales, Rose Mary Michell ^*^and Denis Rodrigue ^*^*


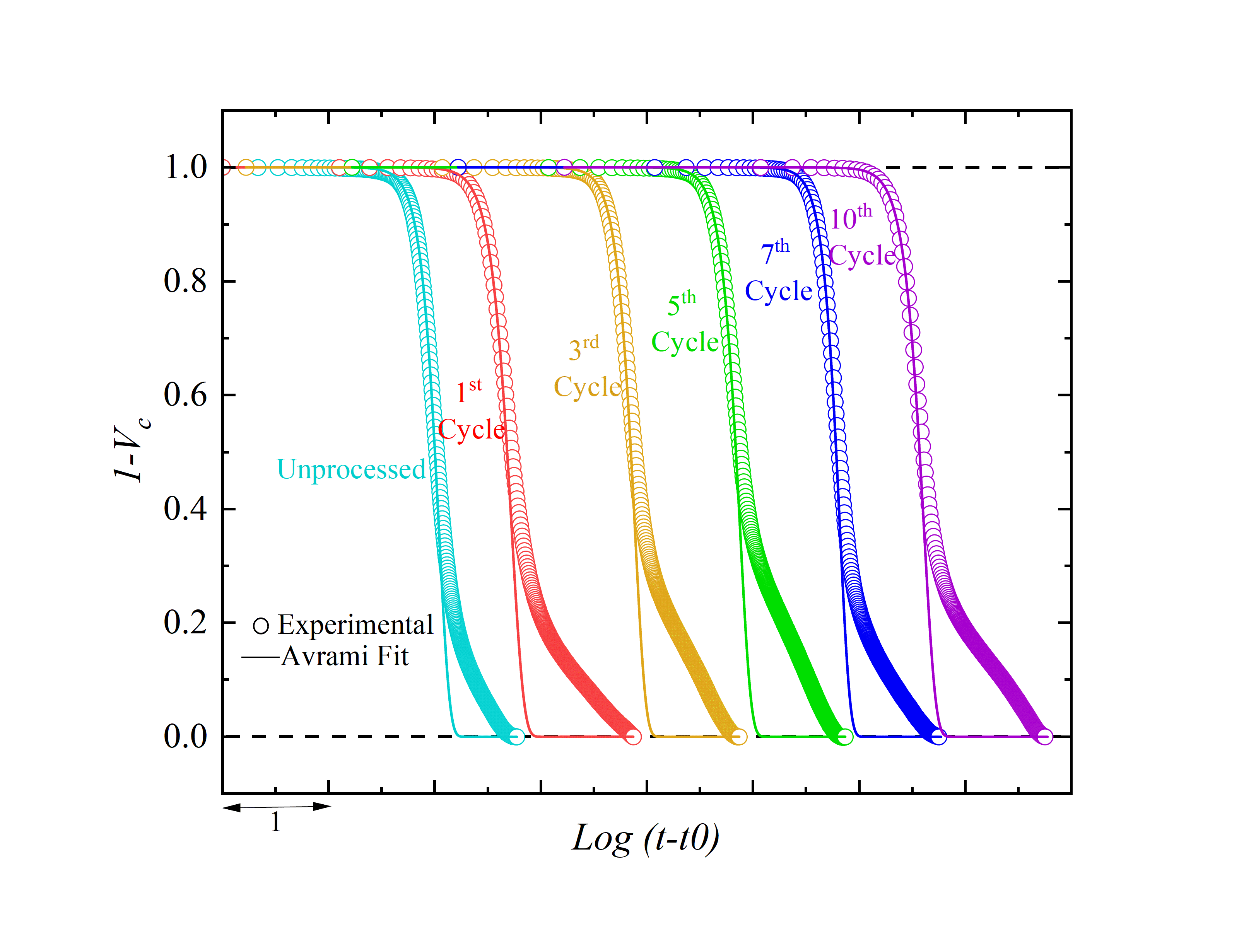

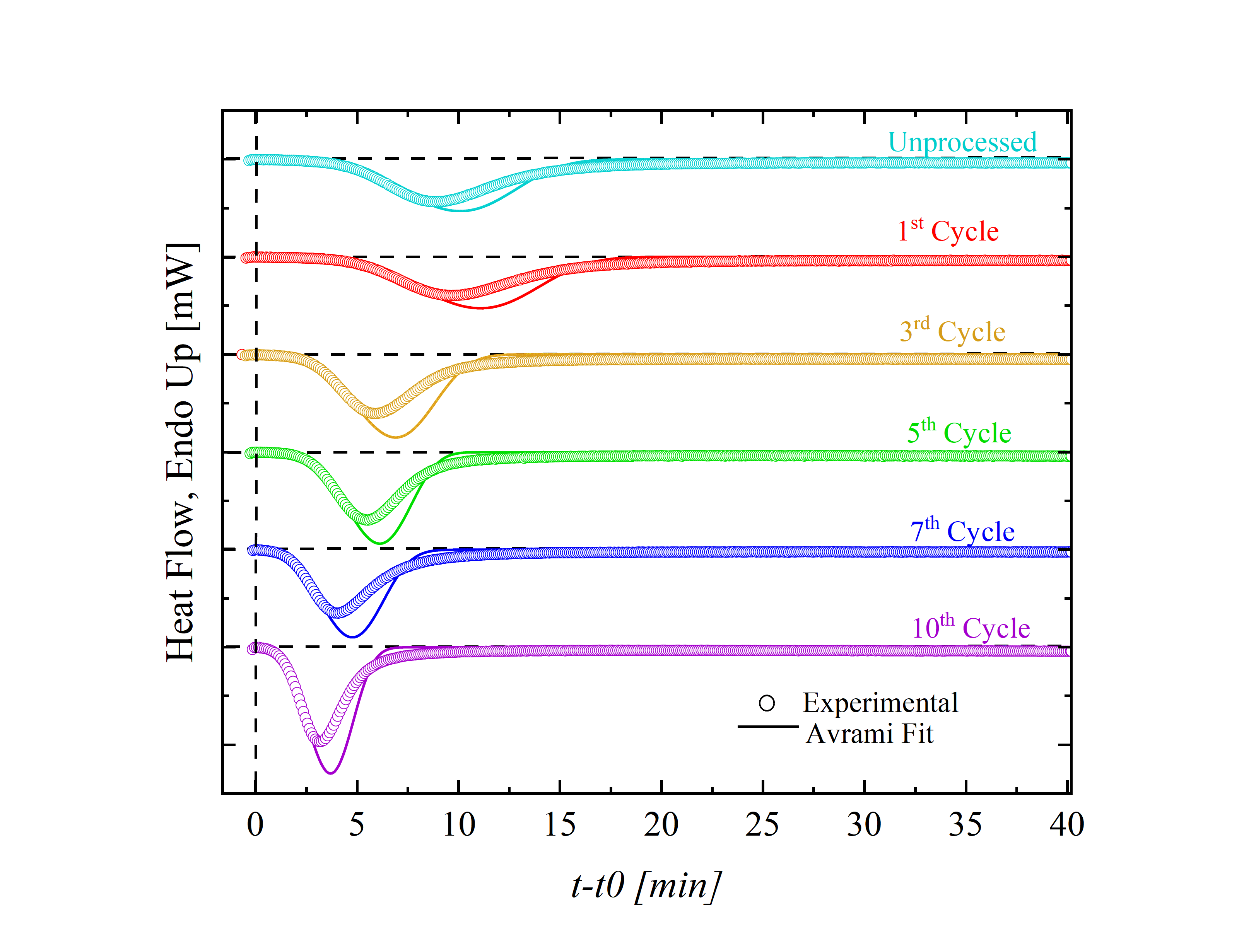

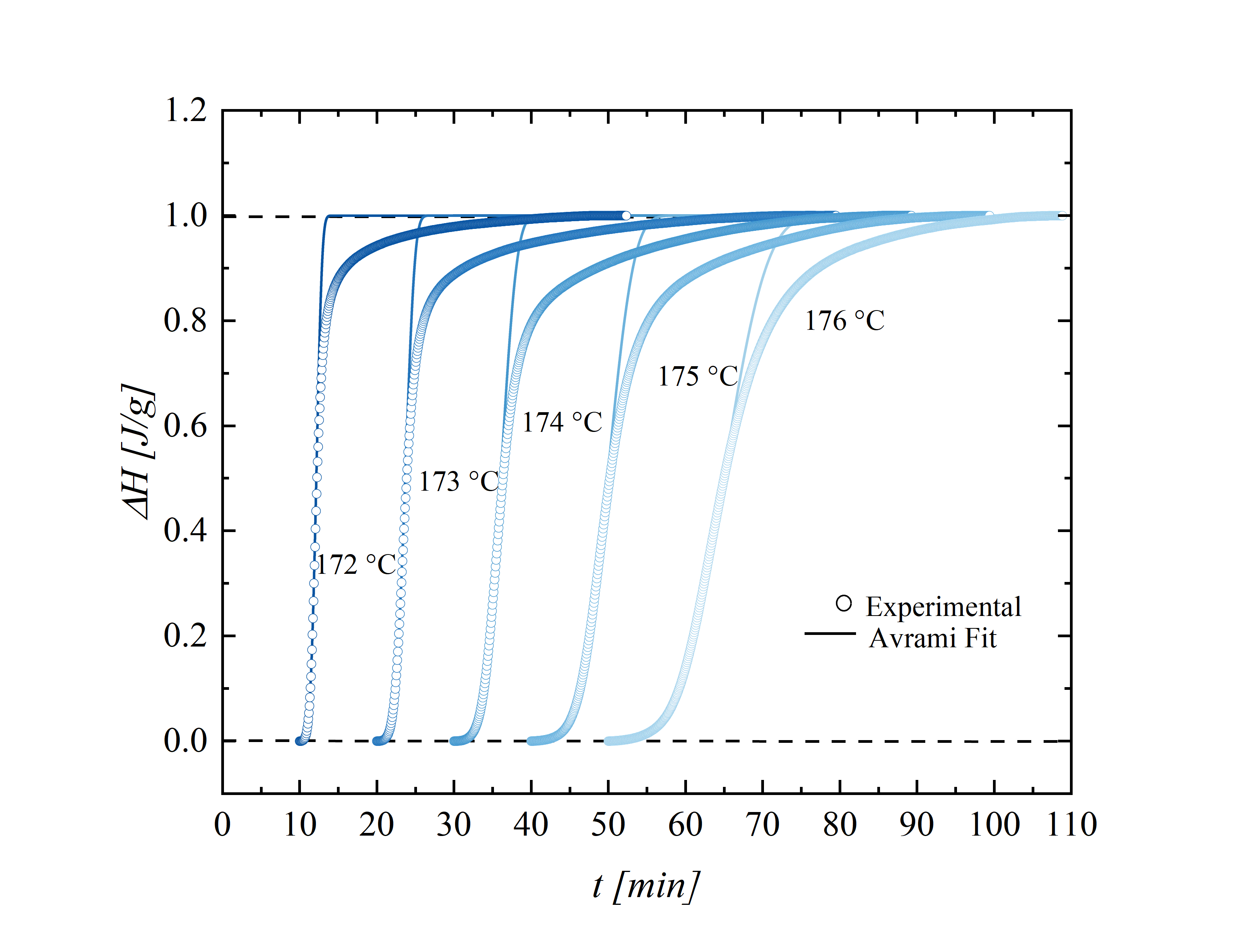

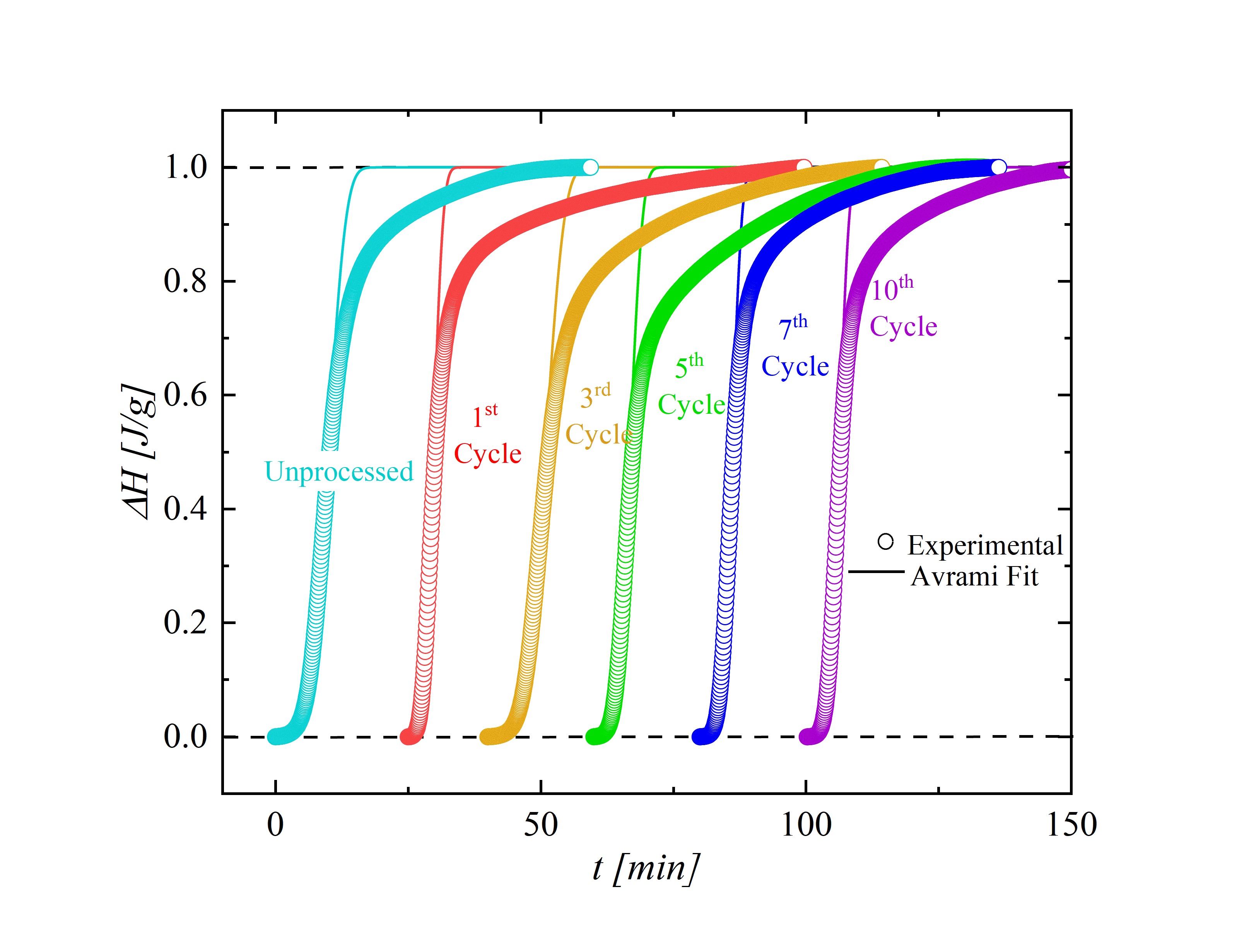


a

d

b

c

**Figure S1.** (a) Variation of the enthalpy of crystallization (*ΔH_c_)* as a function of time (*t*) for the virgin PA 11 at *T_c_* = 172, 173, 174, 175 and 176 °C, (b) Variation of the enthalpy of crystallization (*ΔH_c_*) as a function of time (*t*) for the virgin PA 11 for all reprocessing cycles at *T_c_* = 175 °C, (c) Variation of (1 – *V_c_*) as a function of *log(t − t_0_)* for virgin PA 11 for all reprocessing cycles at *T_c_* = 175 °C, (d) Experimental DSC isotherms and simulated DSC curves by the Avrami equation for virgin PA 11 for all reprocessing cycles at *T_c_* = 175 °C. The lines represent fitting to the Avrami model (Equation (1)) with the parameters of **Table S1**.

**Table S1.** Avrami parameters (*n, K*, $\tau_{theo}^{1/2}, \tau_{exp}^{1/2}$) and their correlation coefficient (R^2^) for the virgin PA 11. The conversion range used for fitting was fixed (3–20%).

| Cycle | *T*  [°C] | *n*  [-] | *K*  [min^-n^] | $\tau_{theo}^{1/2}$  [min] | $\tau_{exp}^{1/2}$  [min] | *R^2^* |
| --- | --- | --- | --- | --- | --- | --- |
| Unprocessed | 172 | 4.31 | 2.28E-02 | 2.21 | 2.32 | 1.0000 |
|  | 173 | 4.43 | 1.96E-03 | 3.76 | 3.97 | 1.0000 |
|  | 174 | 4.33 | 2.63E-04 | 6.18 | 6.52 | 0.9999 |
|  | 175 | 4.30 | 3.41E-05 | 10.07 | 10.44 | 0.9996 |
|  | 176 | 3.91 | 1.68E-05 | 15.17 | 15.36 | 0.9989 |
| 1^st^ | 173 | 3.47 | 7.23E-02 | 1.92 | 2.01 | 1.0000 |
|  | 174 | 3.54 | 1.29E-02 | 3.08 | 3.28 | 1.0000 |
|  | 175 | 3.78 | 1.80E-03 | 4.82 | 5.31 | 0.9999 |
|  | 176 | 3.91 | 3.24E-04 | 7.12 | 7.79 | 1.0000 |
|  | 177 | 3.88 | 7.36E-05 | 10.56 | 11.53 | 0.9999 |
|  | 178 | 3.55 | 4.29E-05 | 15.38 | 16.38 | 0.9995 |
| 3^rd^ | 172 | 3.91 | 8.67E-02 | 1.70 | 1.81 | 1.0000 |
|  | 173 | 4.38 | 6.32E-03 | 2.92 | 3.14 | 0.9999 |
|  | 174 | 4.30 | 1.06E-03 | 4.51 | 4.88 | 0.9999 |
|  | 175 | 4.28 | 1.59E-04 | 7.07 | 7.71 | 1.0000 |
|  | 176 | 4.45 | 1.60E-05 | 11.04 | 11.76 | 0.9999 |
|  | 177 | 4.18 | 5.02E-06 | 16.93 | 17.47 | 0.9993 |
| 5^th^ | 172 | 3.79 | 7.85E-02 | 1.78 | 1.94 | 0.9999 |
|  | 173 | 4.01 | 1.07E-02 | 2.83 | 3.07 | 0.9999 |
|  | 174 | 4.31 | 9.81E-04 | 4.59 | 5.06 | 0.9999 |
|  | 175 | 4.20 | 2.07E-04 | 6.91 | 7.60 | 0.9999 |
|  | 176 | 4.51 | 1.48E-05 | 10.83 | 11.65 | 0.9999 |
|  | 177 | 4.15 | 6.72E-06 | 16.14 | 16.64 | 0.9991 |
| 7^th^ | 172 | 3.75 | 1.42E-01 | 1.53 | 1.61 | 1.0000 |
|  | 173 | 4.09 | 1.42E-02 | 2.59 | 2.78 | 0.9999 |
|  | 174 | 4.49 | 1.29E-03 | 4.06 | 4.33 | 0.9999 |
|  | 175 | 4.59 | 1.73E-04 | 6.09 | 6.47 | 1.0000 |
|  | 176 | 4.20 | 8.13E-05 | 8.63 | 8.95 | 0.9999 |
|  | 177 | 4.53 | 5.15E-06 | 13.58 | 13.97 | 0.9996 |
| 10^th^ | 173 | 3.60 | 1.33E-01 | 1.58 | 1.64 | 1.0000 |
|  | 174 | 3.69 | 2.49E-02 | 2.46 | 2.58 | 1.0000 |
|  | 175 | 3.94 | 3.93E-03 | 3.72 | 4.02 | 1.0000 |
|  | 176 | 4.03 | 9.20E-04 | 5.17 | 5.45 | 0.9999 |
|  | 177 | 4.22 | 1.55E-04 | 7.34 | 7.76 | 0.9999 |
|  | 178 | 4.24 | 3.48E-05 | 10.36 | 10.90 | 0.9999 |


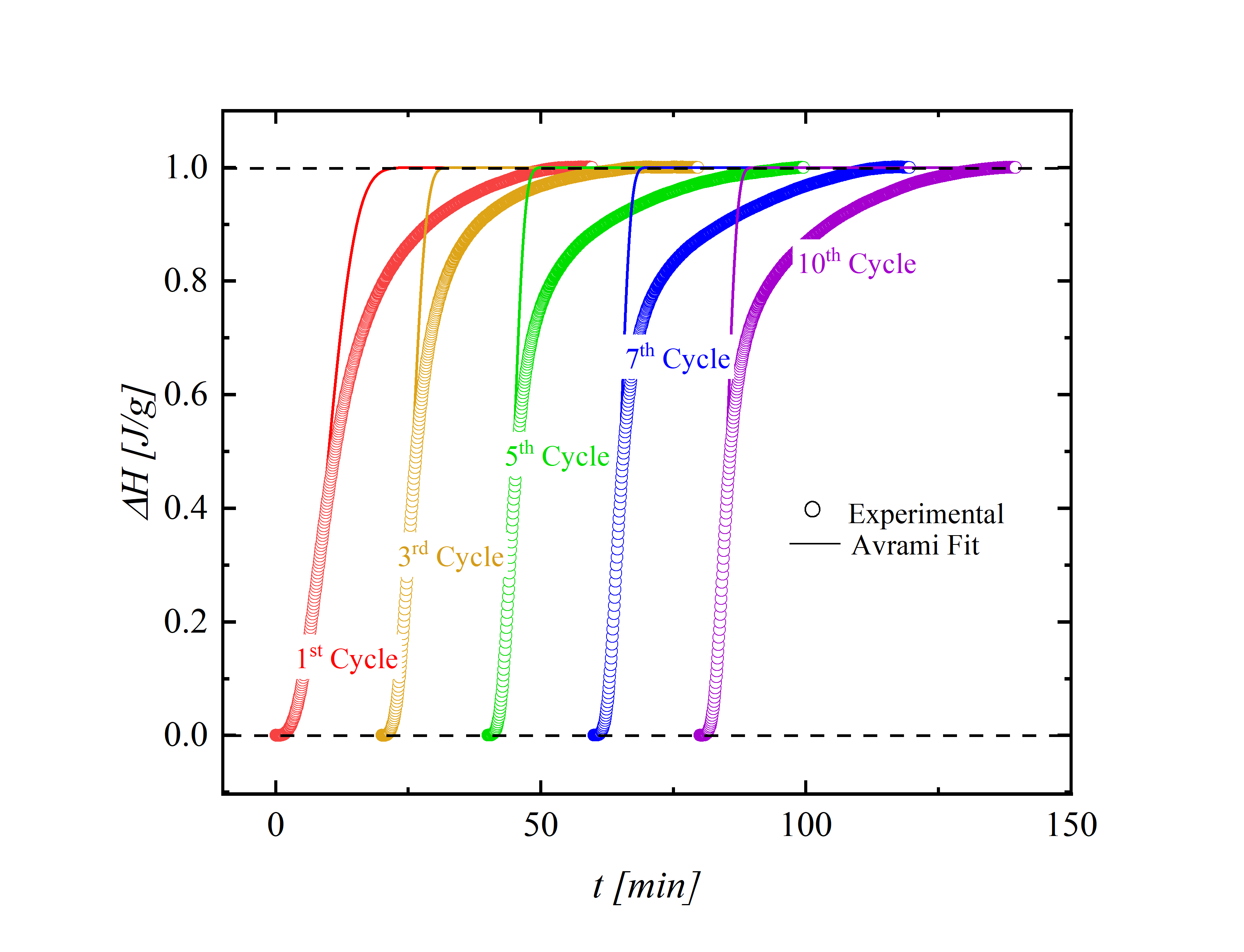

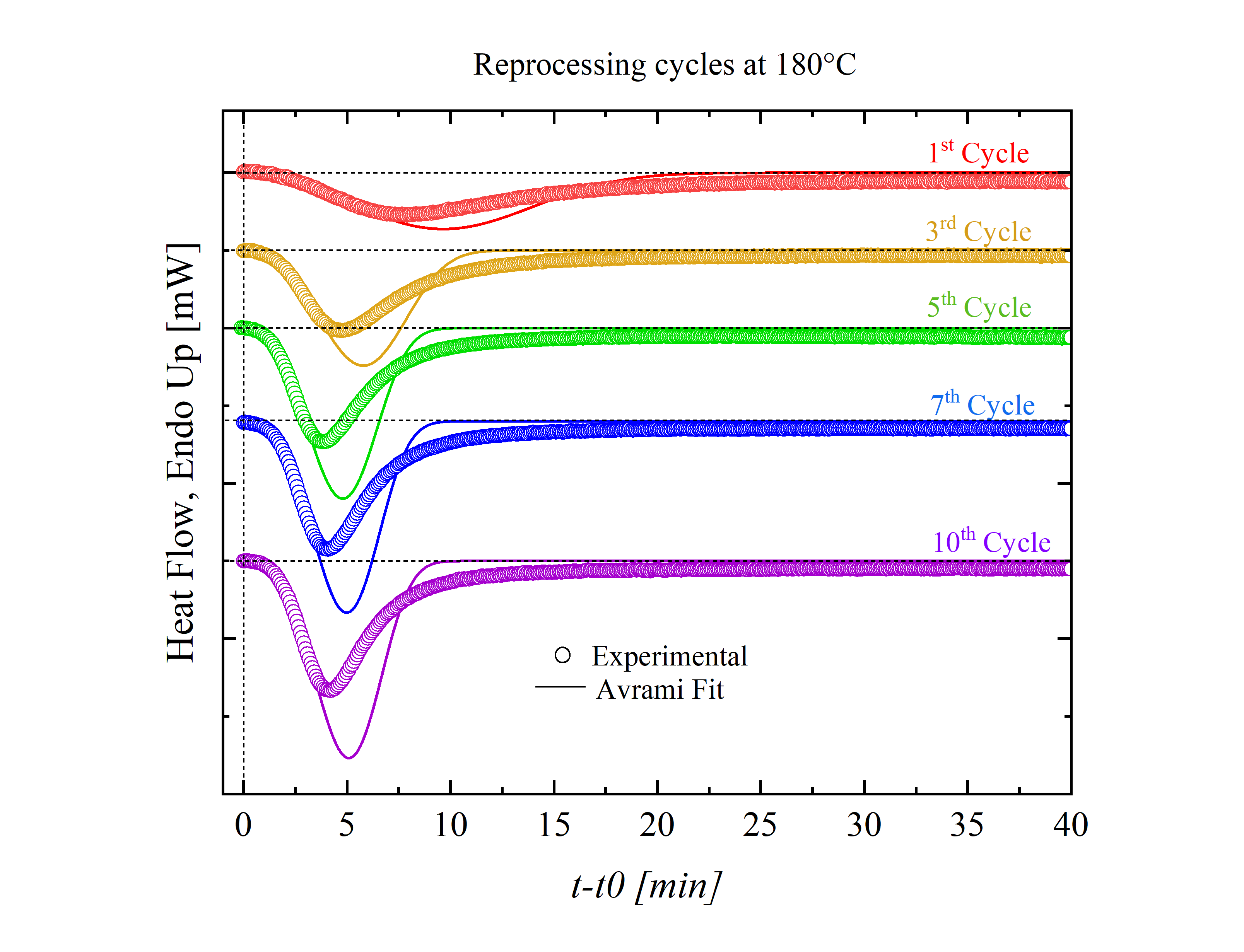

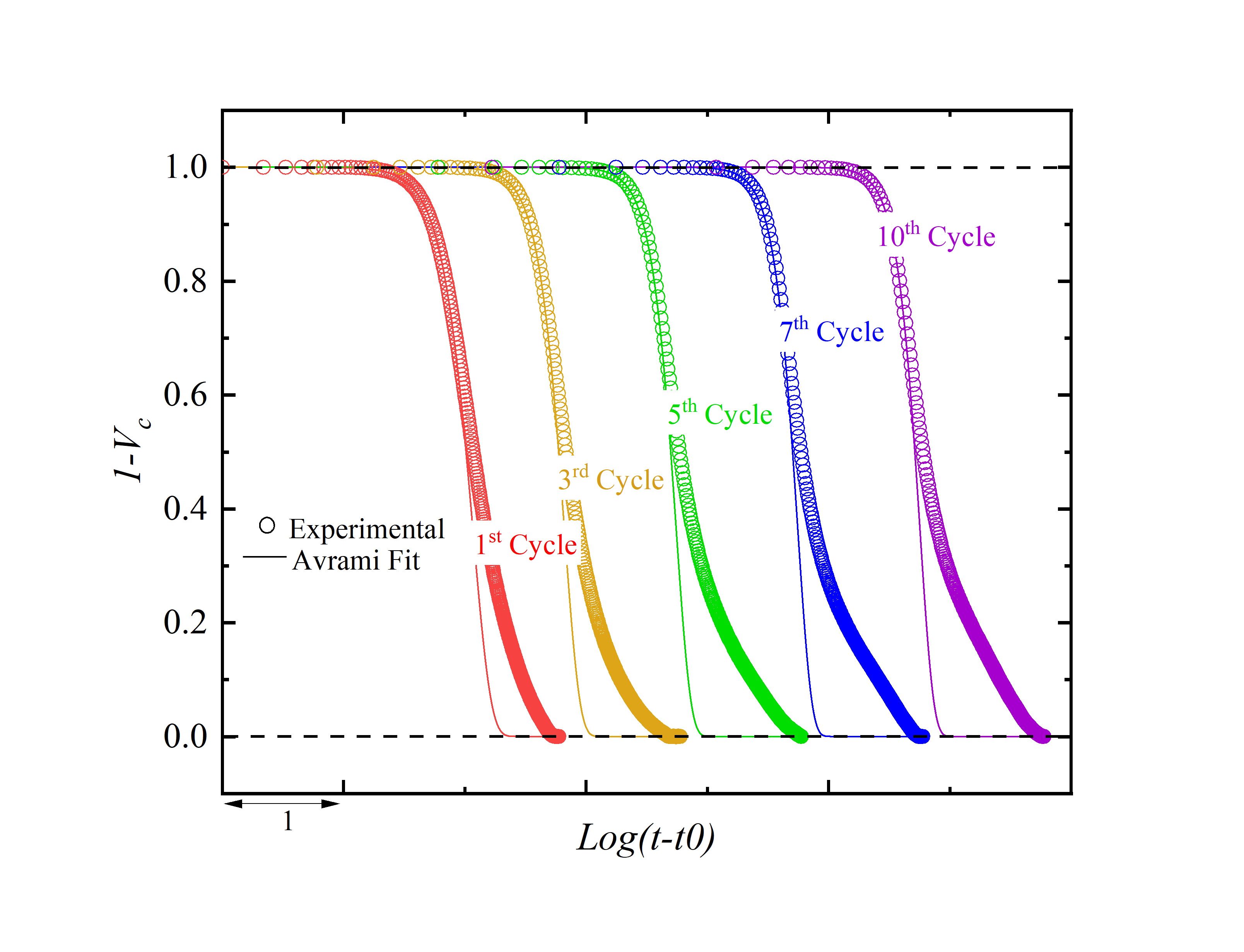

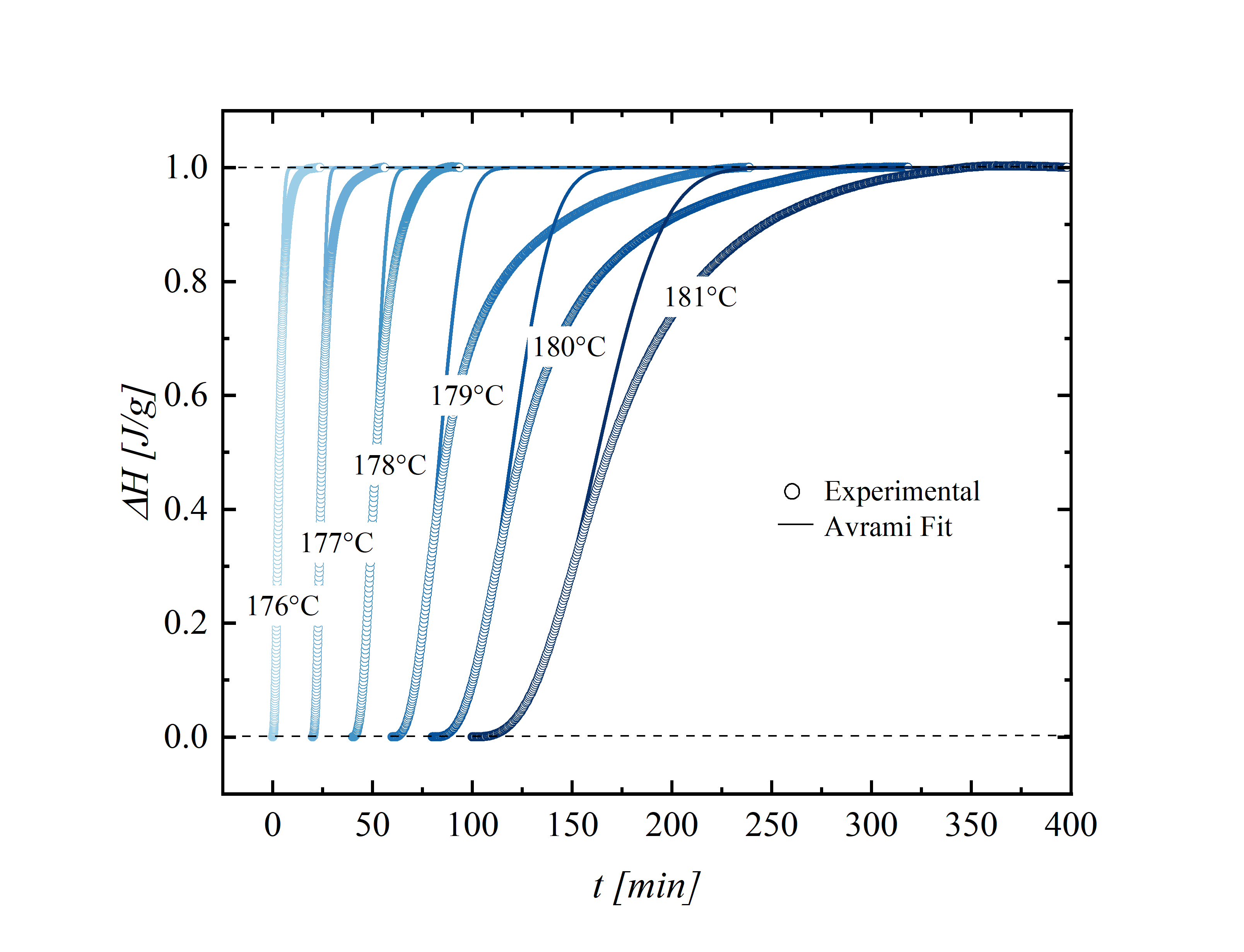


b

d

c

a

**Figure S2.** (a) Variation of the enthalpy of crystallization (*ΔHc*) as a function of time (*t*) for the 1^st^ cycle of post-consumer PA 11 at *T_c_* = 176, 177, 178, 179, 180 and 181 °C, (b) Variation of the enthalpy of crystallization (*ΔHc*) as a function of time (*t*) for the post-consumer PA 11 for all reprocessing cycles at *T_c_* = 180 °C, (c) Variation of (*1 – Vc*) as a function of *log(t − t_0_)* for the post-consumer PA 11 for all reprocessing cycles at *T_c_* = 180 °C, (d) Experimental DSC isotherms and simulated DSC curves by the Avrami equation for post-consumer PA 11 for all reprocessing cycles at *T_c_* = 180 °C. The lines represent fitting to the Avrami model (Equation (1)) with the parameters of **Table S2**.

**Table S2.** Avrami fitting parameters (*n, K*, $\tau_{theo}^{1/2}, \tau_{exp}^{1/2}$) and their correlation coefficient (R^2^) for the post-consumer PA 11. The conversion range used for fitting was fixed (3–20%).

| Cycle | T  [°C] | n  [-] | K  [min^-n^] | $\tau_{theo}^{1/2}$  [min] | $\tau_{exp}^{1/2}$  [min] | R^2^ |
| --- | --- | --- | --- | --- | --- | --- |
| 1^st^ | 176 | 2.42 | 3.78E-02 | 3.32 | 3.53 | 1.0000 |
|  | 177 | 2.59 | 1.42E-02 | 4.50 | 4.92 | 1.0000 |
|  | 178 | 2.73 | 5.94E-03 | 5.94 | 6.23 | 0.9999 |
|  | 179 | 2.78 | 2.04E-03 | 8.12 | 9.43 | 0.9997 |
|  | 180 | 2.90 | 8.27E-04 | 10.22 | 11.65 | 0.9996 |
|  | 181 | 2.91 | 4.05E-04 | 12.90 | 14.20 | 0.9999 |
| 3^rd^ | 177 | 2.92 | 3.88E-02 | 2.68 | 2.93 | 1.0000 |
|  | 178 | 3.16 | 1.12E-02 | 3.69 | 4.25 | 0.9997 |
|  | 179 | 3.22 | 4.60E-03 | 4.75 | 5.53 | 0.9997 |
|  | 180 | 3.43 | 1.56E-03 | 5.93 | 6.77 | 0.9996 |
|  | 181 | 3.57 | 4.72E-04 | 7.71 | 8.97 | 0.9993 |
|  | 182 | 3.61 | 1.68E-04 | 10.01 | 11.60 | 0.9995 |
| 5^th^ | 177 | 3.35 | 4.46E-02 | 2.27 | 2.56 | 0.9998 |
|  | 178 | 3.43 | 1.49E-02 | 3.07 | 3.66 | 0.9994 |
|  | 179 | 3.54 | 6.18E-03 | 3.79 | 4.32 | 0.9997 |
|  | 180 | 3.61 | 2.09E-03 | 4.98 | 5.81 | 0.9996 |
|  | 181 | 3.63 | 7.77E-04 | 6.49 | 7.70 | 0.9995 |
|  | 182 | 3.86 | 1.99E-04 | 8.28 | 9.64 | 0.9994 |
| 7^th^ | 178 | 3.44 | 1.46E-02 | 3.07 | 3.60 | 0.9997 |
|  | 179 | 3.62 | 4.67E-03 | 3.98 | 4.68 | 0.9996 |
|  | 180 | 3.79 | 1.50E-03 | 5.04 | 5.84 | 0.9996 |
|  | 181 | 3.87 | 5.01E-04 | 6.47 | 7.51 | 0.9996 |
|  | 182 | 4.03 | 1.52E-04 | 8.10 | 9.22 | 0.9996 |
|  | 183 | 4.07 | 5.32E-05 | 10.25 | 11.50 | 0.9996 |
| 10^th^ | 178 | 3.58 | 1.08E-02 | 3.20 | 3.70 | 0.9998 |
|  | 179 | 3.72 | 3.88E-03 | 4.04 | 4.65 | 0.9997 |
|  | 180 | 3.86 | 1.26E-03 | 5.13 | 5.99 | 0.9995 |
|  | 181 | 3.87 | 5.16E-04 | 6.44 | 7.35 | 0.9997 |
|  | 182 | 3.91 | 1.97E-04 | 8.06 | 9.17 | 0.9997 |
|  | 183 | 3.94 | 6.90E-05 | 10.38 | 11.68 | 0.9998 |


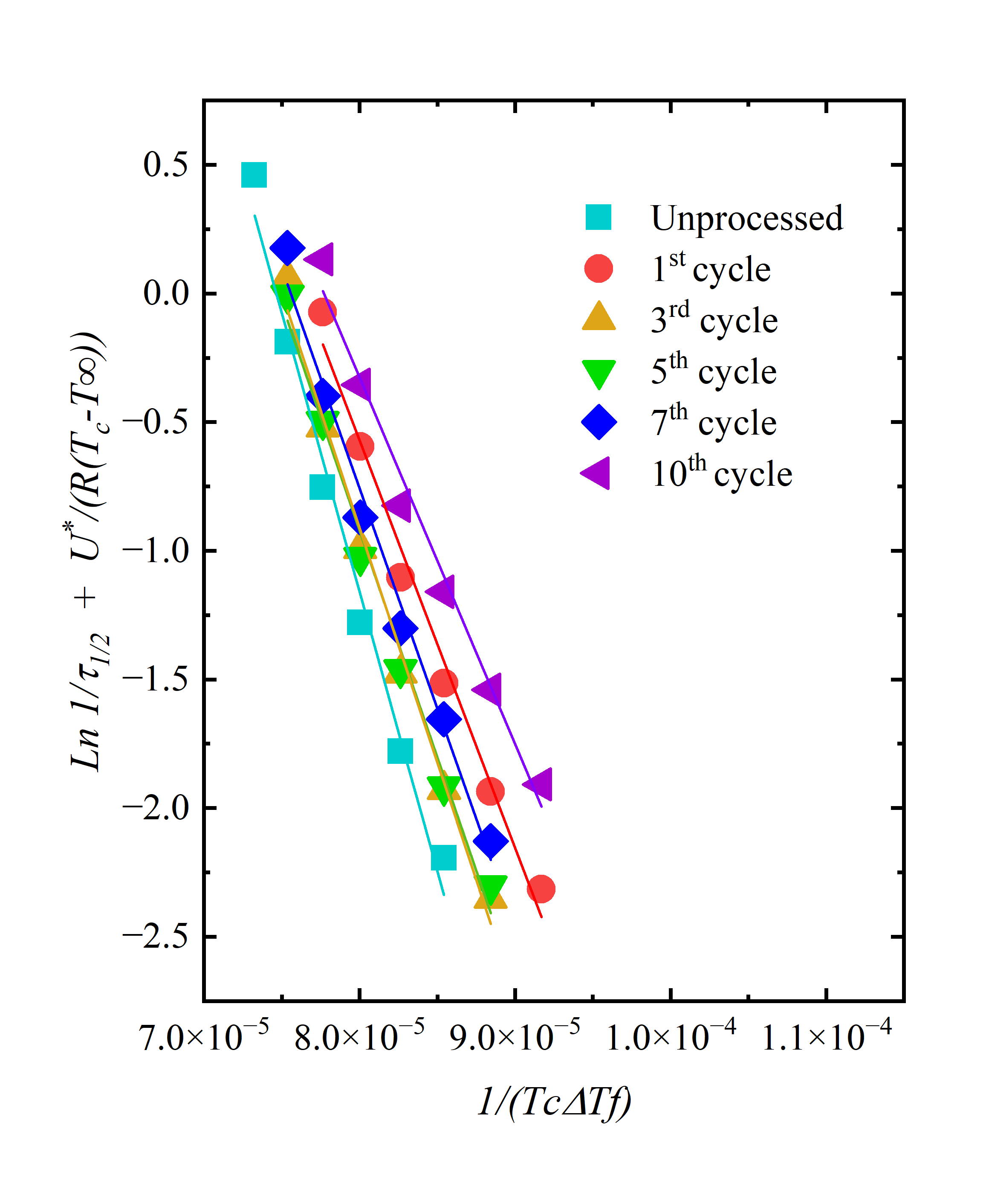

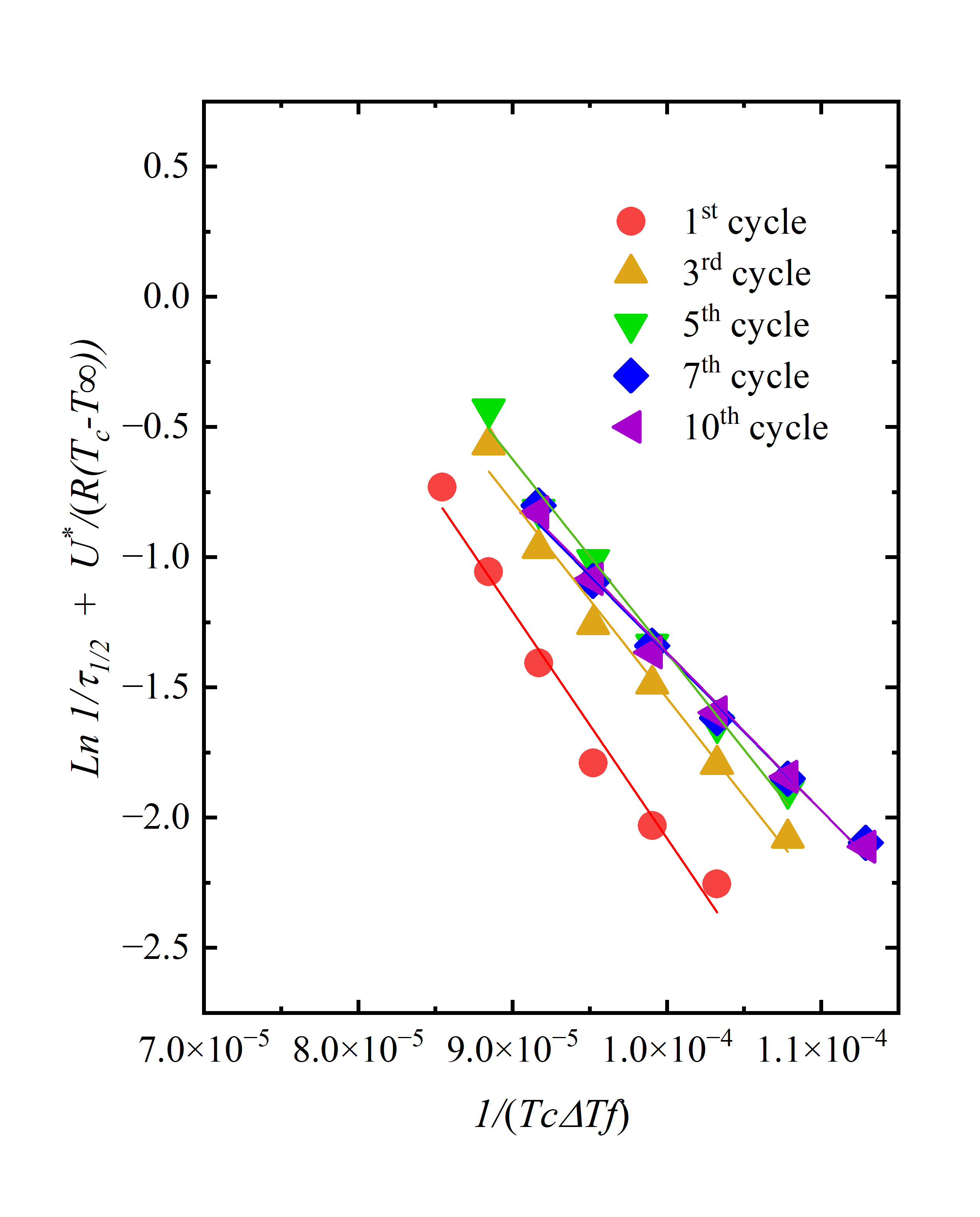


a

b

**Figure S3.** Plots of *Ln(1/τ_1/2_) + U*/R (T_c_ − T_∞_)* as a function of *(1/T_c_ΔT_f_)* for: (a) virgin PA 11 and (b) post-consumer PA 11.


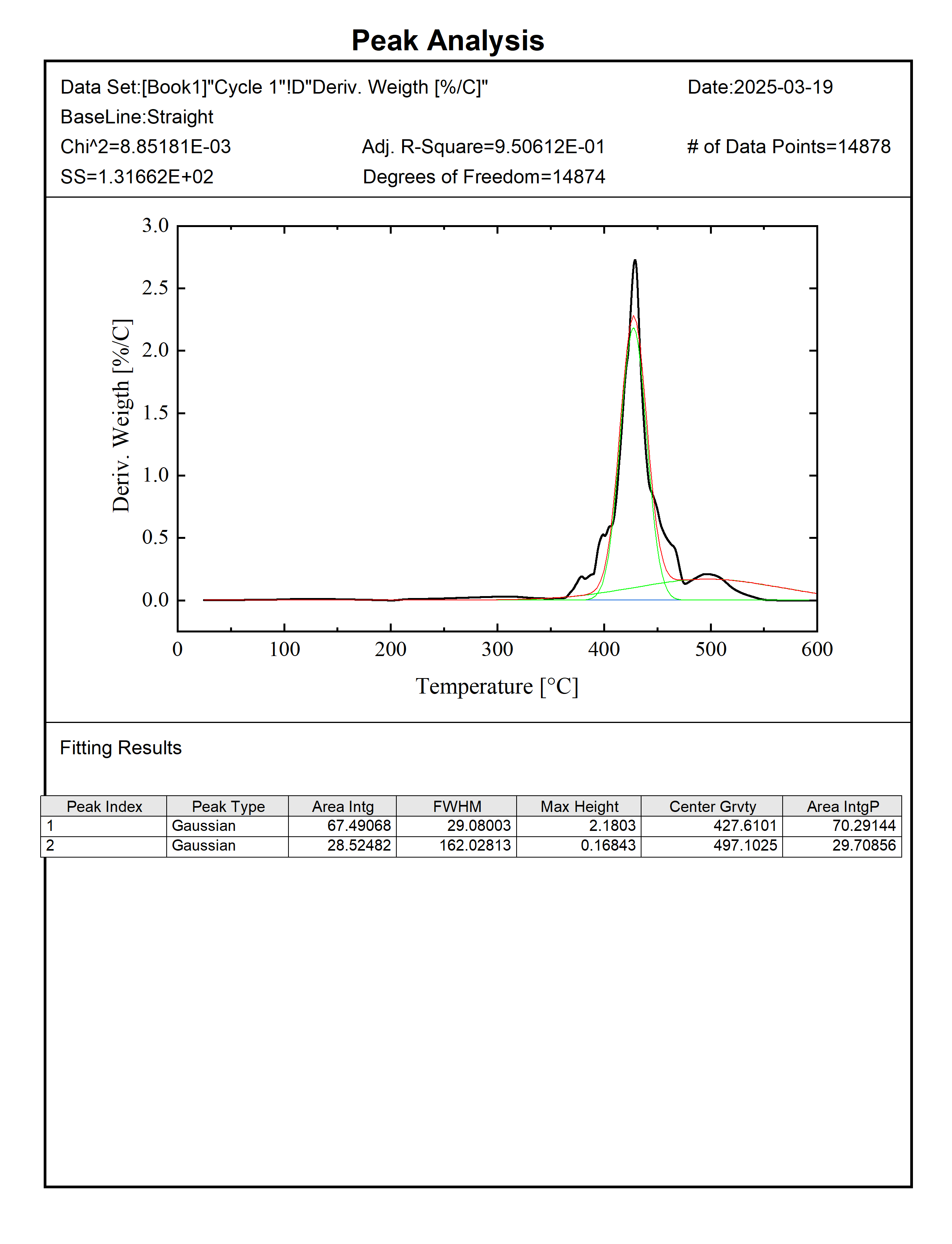

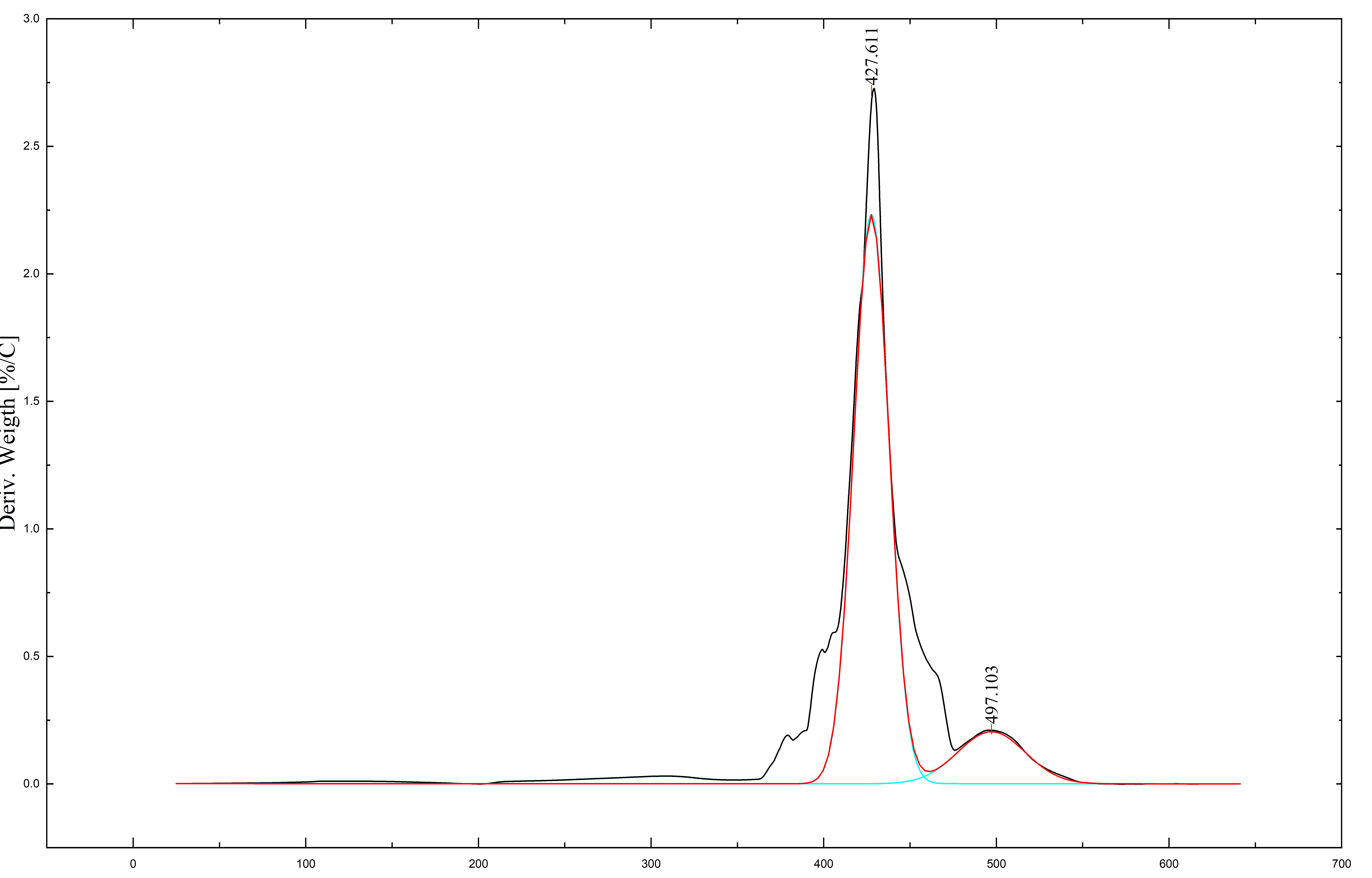


Peak_1_= 427.601 °C

Peak_2_= 497.103 °C


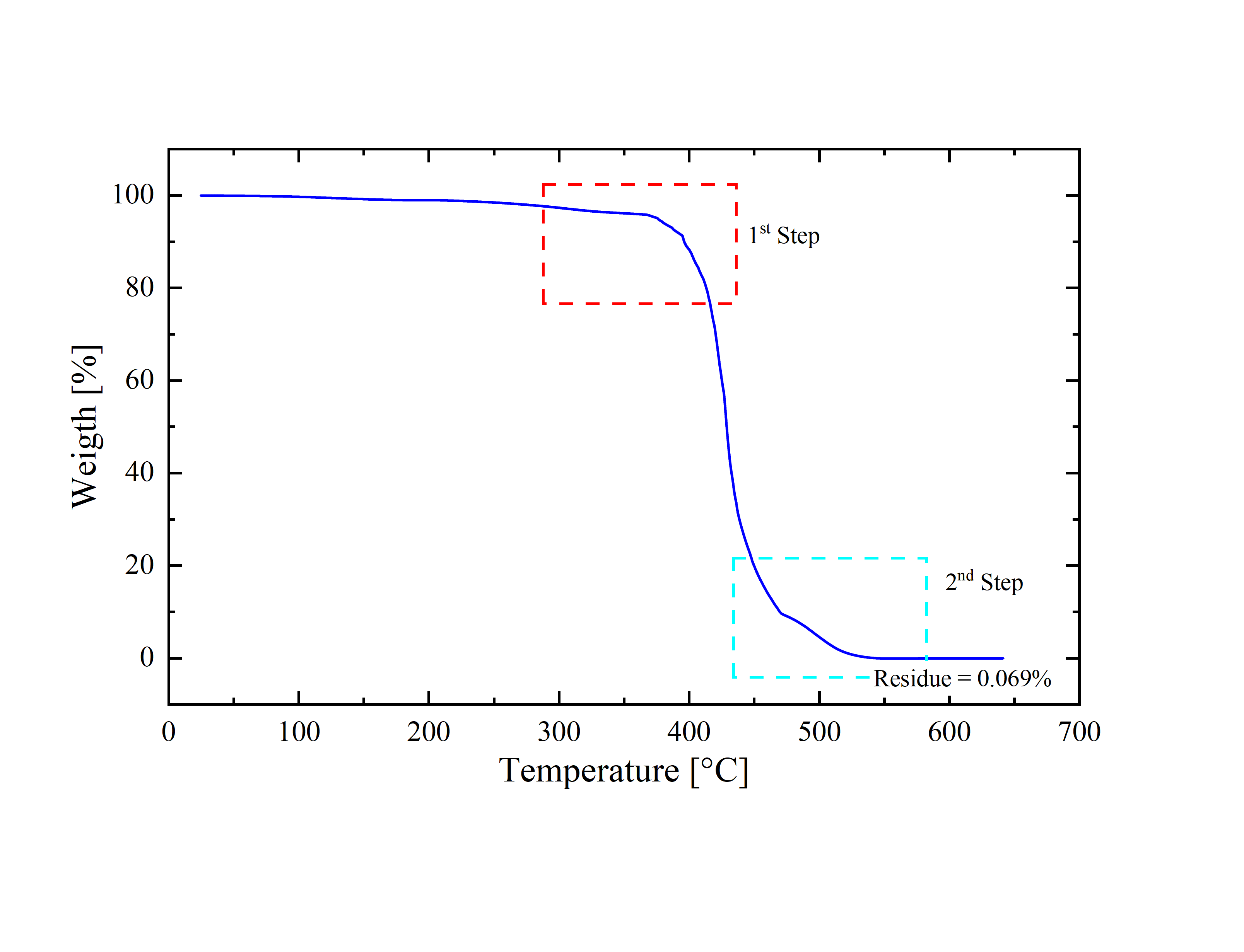


b

a

**Figure S4.** TGA curves for post-consumer PA 11: (a) weight as a function of temperature from 20 to 650°C at a heating rate of 5°C/min under an air atmosphere and (b) the derivative (DTG) curve deconvoluted into two peaks corresponding to PA 11 and LDPE, respectively.
